# *De novo* GTP Biosynthesis Is Critical for Virulence of the Fungal Pathogen *Cryptococcus neoformans*

**DOI:** 10.1371/journal.ppat.1002957

**Published:** 2012-10-11

**Authors:** Carl A. Morrow, Eugene Valkov, Anna Stamp, Eve W. L. Chow, I. Russel Lee, Ania Wronski, Simon J. Williams, Justine M. Hill, Julianne T. Djordjevic, Ulrike Kappler, Bostjan Kobe, James A. Fraser

**Affiliations:** 1 Australian Infectious Diseases Research Centre, University of Queensland, Brisbane, Queensland, Australia; 2 School of Chemistry and Molecular Biosciences, University of Queensland, Brisbane, Queensland, Australia; 3 MRC Laboratory of Molecular Biology, Cambridge, United Kingdom; 4 Division of Chemistry and Structural Biology, Institute for Molecular Bioscience, University of Queensland, Brisbane, Queensland, Australia; 5 Centre for Advanced Imaging, University of Queensland, Brisbane, Queensland, Australia; 6 Centre for Infectious Diseases and Microbiology, Westmead Millennium Institute, University of Sydney at Westmead Hospital, Sydney, New South Wales, Australia; University of Toronto, Canada

## Abstract

We have investigated the potential of the GTP synthesis pathways as chemotherapeutic targets in the human pathogen *Cryptococcus neoformans*, a common cause of fatal fungal meningoencephalitis. We find that *de novo* GTP biosynthesis, but not the alternate salvage pathway, is critical to cryptococcal dissemination and survival *in vivo*. Loss of inosine monophosphate dehydrogenase (IMPDH) in the *de novo* pathway results in slow growth and virulence factor defects, while loss of the cognate phosphoribosyltransferase in the salvage pathway yielded no phenotypes. Further, the *Cryptococcus* species complex displays variable sensitivity to the IMPDH inhibitor mycophenolic acid, and we uncover a rare drug-resistant subtype of *C. gattii* that suggests an adaptive response to microbial IMPDH inhibitors in its environmental niche. We report the structural and functional characterization of IMPDH from *Cryptococcus*, revealing insights into the basis for drug resistance and suggesting strategies for the development of fungal-specific inhibitors. The crystal structure reveals the position of the IMPDH moveable flap and catalytic arginine in the open conformation for the first time, plus unique, exploitable differences in the highly conserved active site. Treatment with mycophenolic acid led to significantly increased survival times in a nematode model, validating *de novo* GTP biosynthesis as an antifungal target in *Cryptococcus*.

## Author Summary

The fungal pathogen *Cryptococcus neoformans* is responsible for up to a million deaths annually, and the currently available antifungal medicines used to combat this infection are limited and patently inadequate. We have investigated the GTP biosynthesis pathway as a new drug target, a pathway that has been a chemotherapy target in humans for many years. We found that inhibiting or deleting one specific enzyme in this pathway, IMP dehydrogenase, was extremely effective at weakening *Cryptococcus* as a pathogen or killing it outright. Intriguingly, we isolated an extremely rare *Cryptococcus* isolate that was naturally drug resistant, suggesting that microbial competitors of *Cryptococcus* may also use this strategy to out-compete it in the wild. By comparison of the subtype responsible for the majority of infections worldwide with this rare drug resistant form, we have characterized the enzyme's structure and mechanism, revealing a number of strategies to develop more potent and specific anti-IMP dehydrogenase drugs for *Cryptococcus*. Treating nematodes grown on *Cryptococcus* with the inhibitors led to longer survival times for the worms, validating our strategy.

## Introduction

Fungal infections of humans are highly refractive to pharmacological intervention due to the similarities in eukaryotic cell physiology. The limited array of fungal cell-specific features has therefore been the focus of antifungal drug research for many years, with the fungal cell wall and cell membrane being primary targets. Recent studies exploring potential drug targets in fungal genomes have found a surprisingly small number of essential targets with little identity to a human homologue [1]–[4]. An alternate approach to targeting fungal-specific components is therefore to instead target shared proteins that are well characterized in both the host and pathogen, and exploit more subtle differences between the two. This approach is exemplified by the novel antifungal sordarin and its derivatives [5], [6].

One of the leading life-threatening fungal infections worldwide is cryptococcal meningitis caused by *Cryptococcus neoformans*, a pathogen that infects primarily immunocompromised individuals, and its sister species *Cryptococcus gattii*, which generally infects immunocompetent individuals [7]. The estimated annual global incidence of cryptococcal meningitis is estimated to be 1.1 million cases annually, causing ∼624,000 deaths per year, mostly in areas with high HIV rates, such as sub-Saharan Africa. These are alarming numbers for a pathogen whose treatment regimen has not altered significantly in over a decade [8]–[10]. Treatment of systemic fungal infections predominantly relies on a small group of antifungals comprising azoles, polyenes, echinocandins and the antimetabolite flucytosine. Numerous problems exist with these treatments however, including their notoriously variable efficacy across the limited spectrum of human fungal pathogens, high cost and toxicity, a frequent requirement for hospitalization, and emerging drug resistance [10], [11]. The design of new classes of effective, readily available and affordable antifungals is therefore a matter of urgency.

Rational drug design was pioneered in the purine metabolic pathway, a conserved series of processes responsible for providing the cell with a ready supply of ATP and GTP as both an energy source and for critical cellular processes including replication, transcription, translation and signal transduction. This pathway has continued to serve as a fertile source of therapeutic agent development for over fifty years [12], and growing evidence supports it as a potential source of effective antifungal targets. Disruption of *de novo* ATP or GTP biosynthesis genes in *Candida albicans* and *Aspergillus fumigatus* leads to complete avirulence in mammalian models [13]–[15]. In *Cryptococcus*, mutations that globally affect ATP and GTP biosynthesis lead to attenuated or complete loss of virulence *in vivo*, as well as general growth defects and impaired virulence factor expression [16], [17].

Two key enzymes supplying guanine nucleotides to a cell are inosine monophosphate (IMP) dehydrogenase (IMPDH), the rate-limiting catalyst and first committed step of *de novo* GTP biosynthesis, and hypoxanthine-xanthine-guanine phosphoribosyltransferase (HXGPRT), responsible for recycling purine nucleobases into nucleoside monophosphates in the GTP and ATP salvage pathways. As a key metabolic enzyme, IMPDH is highly expressed in proliferating cells and has become a major target of immunosuppressive and antiviral chemotherapy, and has attracted great interest as an anticancer, antiprotozoal, antibacterial and antifungal target [18]–[21]. Four IMPDH inhibitors are currently approved for treatments: the immunosuppressants mycophenolic acid (MPA) and mizoribine, the anticancer agent tiazofurin, and the antiviral ribavirin. There are significant structural and functional differences between microbial and human IMPDHs, suggesting that species-specific inhibitors of key metabolic pathways hold considerable potential as novel therapeutics [19], [21]–[23]. In this study we have investigated the potential of the GTP biosynthesis pathway and the enzymes IMPDH and HXGPRT as candidate antifungal targets using genetic, structural and functional approaches to validate purine metabolism as a viable chemotherapeutic target in *C. neoformans*.

## Results

### Purine metabolism in *Cryptococcus* lacks several canonical pathway elements

**Figure 1:**
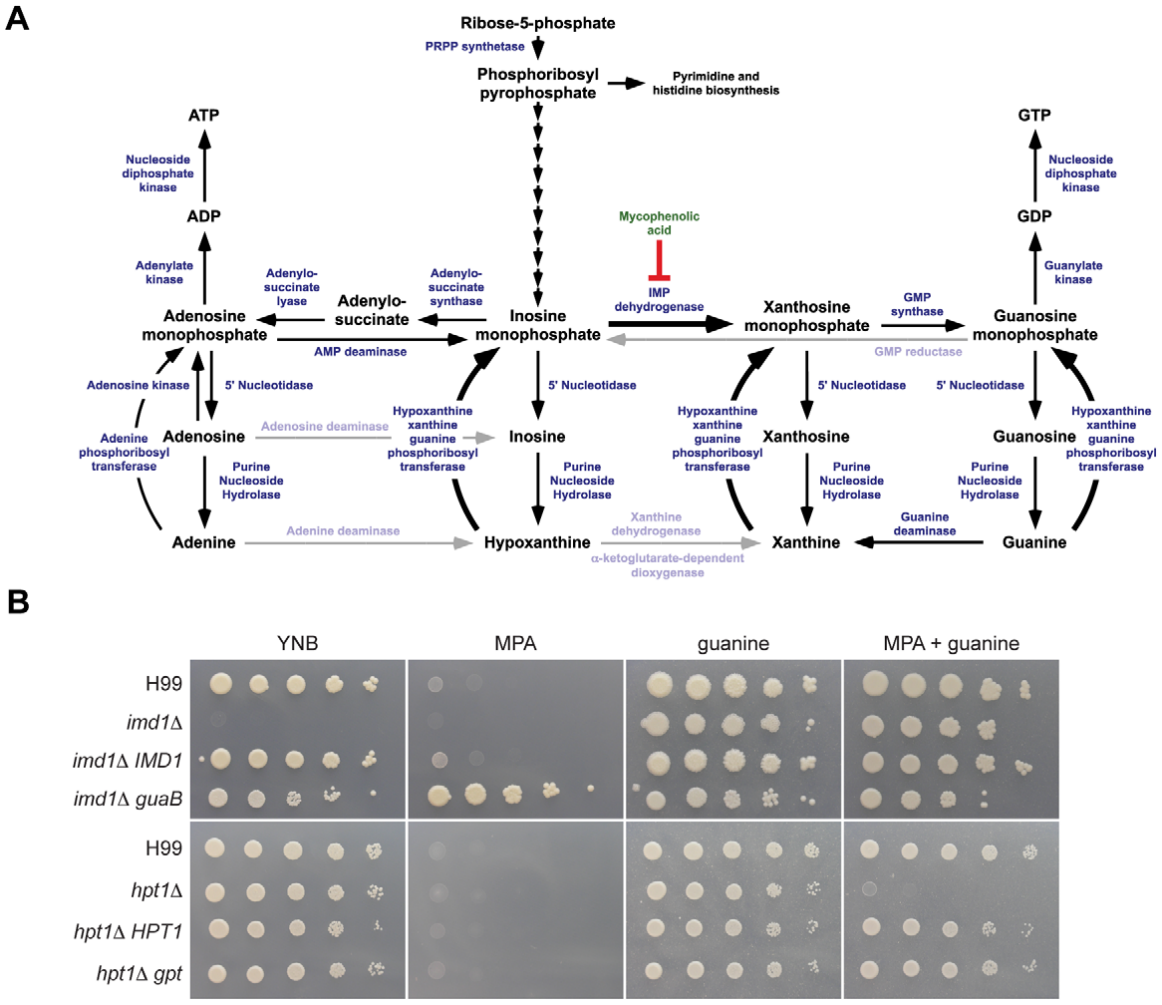
Components of the purine metabolic pathway in *Cryptococcus*. (A) BLASTp analysis using *S. cerevisiae* orthologs reveals that the majority of the components of the canonical purine biosynthetic pathway are present in the genome of *C. neoformans* var. *grubii*. Enzymes or activities missing are greyed. (B) 10-fold serial dilutions of indicated strains were spotted onto YNB medium supplemented with specified purines (1 mM) and/or MPA (5 µg/mL) and incubated for two days at 30°C. The *imd1*Δ deletion strain is an auxotroph; MPA mimics the effect of an *imd1*Δ deletion, while *E. coli* guaB is highly resistant to MPA. A phenotype for the *hpt1*Δ strain is only observed in the presence of MPA.

Unlike the purine-rich pigeon guano natural environment of *C. neoformans*, the human central nervous system inhabited during systemic infection is purine-poor [24], suggesting that during infection *de novo* purine synthesis could be important for cell survival. A bioinformatic survey of the available *C. neoformans* and *C. gattii* genomes to identify components of the purine biosynthetic pathway identified *Cryptococcus* homologs of most genes of the canonical purine pathway (Figure 1A), with the exception of adenosine deaminase, adenine deaminase, and GMP reductase. As previously reported [25], xanthine dehydrogenase is also absent but a potential equivalent, an α-ketoglutarate-dependent dioxygenase, is present. Each gene identified is present as a single copy, including those encoding two key components of the GTP biosynthetic pathway: IMPDH required for *de novo* GTP synthesis (*IMD1*) and a phosphoribosyltransferase (PRTase) required for the GTP salvage pathway (*HPT1*).

### IMPDH is essential for *de novo* GTP biosynthesis

IMPDH performs the rate-limiting, first step in *de novo* GTP biosynthesis, the NAD^+^-dependent conversion of inosine monophosphate (IMP) to xanthosine monophosphate (XMP) *via* a two-step oxidation and hydrolysis reaction. The reaction mechanism is complex and involves a large conformational change mid-reaction, which a number of inhibitors exploit [26]–[30]. To determine if the *C. neoformans IMD1* gene encodes a *bona fide* IMPDH, we deleted it in the well-characterized *C. neoformans* var. *grubii* strain H99. The *imd1*Δ strain was a guanine auxotroph and could not grow on minimal medium (Figure 1B). Supplementation of the medium with exogenous guanine restored growth, which we later showed was *via* a salvage pathway. Introduction of the *E. coli* IMPDH *guaB* into the deletion mutant fully restored growth on minimal medium, confirming that the *IMD1*-encoded enzyme performs the conversion of IMP to XMP (Figure 1B). Finally, the *imd1*Δ phenotype could be mimicked by the addition of the IMPDH inhibitor MPA to the growth medium; both the wild-type and *IMD1* complemented strains were equally sensitive to MPA at low concentrations (5 µg/mL). This phenotype is abolished when the media is supplemented with guanine, as the salvage pathway is able to bypass the block at IMPDH.

### *imd1*Δ rescue *via* purine salvage is mediated by phosphoribosyltransferase Hpt1

Purines present in both the bird guano ecological niche of *Cryptococcus* and within the human host are potential substrates of a GTP salvage pathway. Purine salvage is mediated by PRTase enzymes, which catalyze the transfer of a ribose 5-phosphate-derived phosphoribosylpyrophosphate group to guanine or other purine nucleobases [31]. Upon deletion of the identified *HPT1* gene in strain H99, the mutant exhibited wild-type growth on rich and minimal media (Figure 1B). MPA-mediated growth inhibition was abolished by the presence of guanine in all strains but the *hpt1*Δ mutant, demonstrating that Hpt1 mediates salvage of guanine (Figure 1B). Heterologous expression of the *E. coli* PRTase *gpt* in the *hpt1*Δ mutant restored wild-type growth on guanine, confirming the role of the *C. neoformans* gene as a PRTase. Further testing revealed that Hpt1 could accept hypoxanthine, xanthine and guanine as substrates, defining the enzyme as a hypoxanthine-xanthine-guanine phosphoribosyltransferase (HXGPRT) (Figure S1).

### Loss of IMPDH delays synthesis of the cryptococcal polysaccharide capsule and melanin

**Figure 2:**
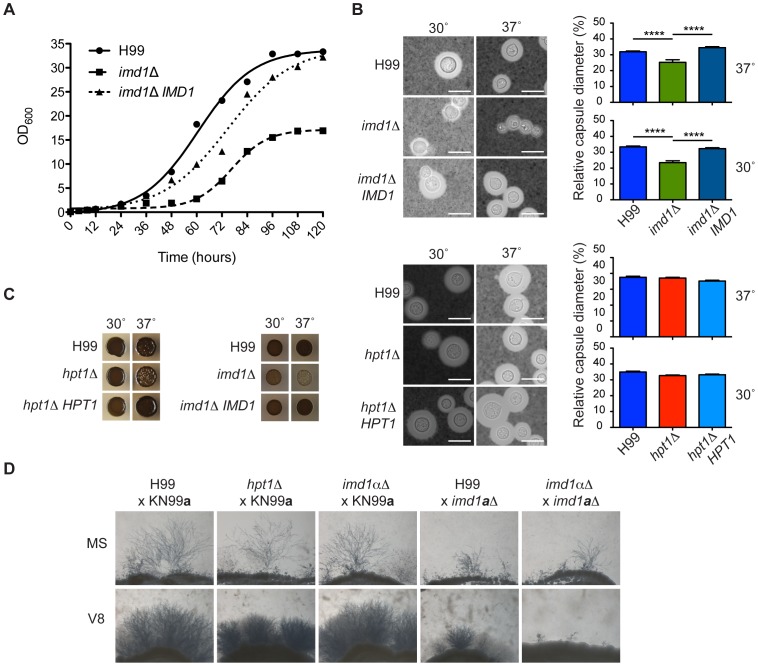
Contribution of purine biosynthesis genes to the virulence component of *Cryptococcus*. (A) Strains were grown for five days in RPMI 1640 media supplemented with 10% serum and monitored spectrophotometrically at 600 nm. (B) *C. neoformans* capsule biosynthesis was examined by growth in RPMI 1640 media plus 10% serum for 16 hours at 30°C and visualized using India ink; the halo of clearance denotes the capsule. Scale bar is 10 µm. Capsular diameter was measured relative to cellular diameter using CellProfiler image analysis software. Bars denote the average relative diameter from three biological replicates. Standard error bars are shown; *p*<0.05 *; *p*<0.01 **; *p*<0.001 ***; *p*<0.0001 ****. (C) Melanin production was determined on *Cryptococcus* l-DOPA melanization plates incubated for two days. (D) Mating proficiency was assayed by co-culturing strains on Murashige-Skoog or V8 medium in the dark for two weeks at 25°C. Magnification is 40×. All assays involving the *imd1*Δ strain were supplemented with 1 mM guanine.

As GTP biosynthesis is crucial to many cellular processes, we sought to determine if guanine auxotrophy affected pathogenicity *via* effects on virulence factor production. Despite addition of guanine, the wild-type phenotype was not completely restored in the *imd1*Δ mutant, which exhibited slow growth at 30°C and was unable to grow on rich media. Accordingly, in a growth curve assay the *imd1*Δ mutant had an extended lag phase of almost 48 hours compared to wild-type and reached a much lower final cell density (Figure 2A). In capsule-inducing conditions the *imd1*Δ strain showed a significantly smaller capsule after 24 hours at both 30°C and 37°C (Figure 2B), approximately one-third and one-quarter smaller, respectively. Capsule size after extended growth (≥96 hours) was similar to wild-type (data not shown). On solid melanization media the *imd1*Δ mutant demonstrated reduced melanin production at both 30°C and human body temperature of 37°C (Figure 2C), although this defect was not apparent in liquid l-DOPA medium (Figure S2). Elaboration of hyphae was notably attenuated in matings involving an *imd1*Δ mutant created in an isogenic *MAT* **a** mating-type background, while the *imd1*Δ mutant in the *MAT*α mating-type background was unaffected (Figure 2D). In contrast to the pleiotropism of the *imd1*Δ mutation, the *hpt1*Δ mutant showed no apparent defect in growth at human body temperature, capsule production, or melanization at either 30°C or 37°C, and loss of Hpt1 also appeared to have no effect on hyphal filamentation during mating (Figure 2B, C, D and data not shown).

### The GTP salvage pathway is dispensable for *C. neoformans* virulence in two animal models

**Figure 3:**
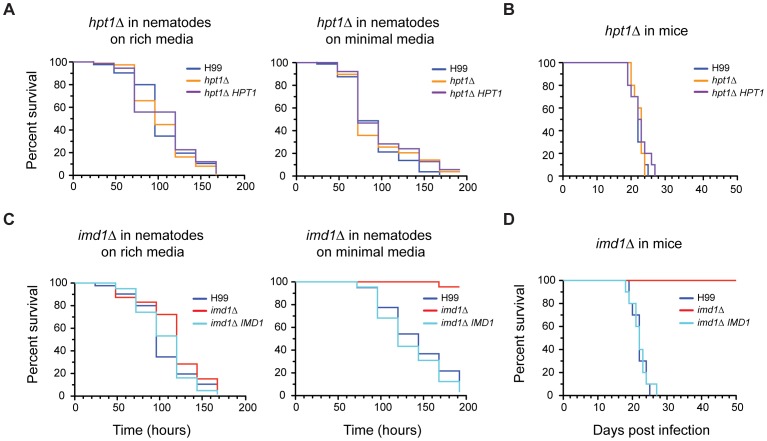
Virulence of *imd1*Δ and *hpt1*Δ in invertebrate and mammalian host systems. (A) Survival of Bristol N2 strain nematodes co-cultured with indicated *Cryptococcus* strains cultured on rich Brain Heart Infusion medium or minimal YNB medium at 25°C. (B) Survival of female BALB/c mice infected with 5×10^5^ cells *via* nasal inhalation. (C) Virulence of indicated *Cryptococcus* strains co-cultured with nematodes on rich and minimal medium at 25°C. (D) Virulence of the *imd1*Δ mutant in BALB/c mice infected with 5×10^5^ cells *via* nasal inhalation. Virulence was determined using Kaplan-Meier survival analysis with statistical significance determined using a log-rank test. Survival times were not significantly different for the *hpt1*Δ strain in either model. *imd1*Δ was significantly attenuated in the nematode model on minimal medium (*p*<0.0001) and in the murine model (*p*<0.0001).

GTP salvage pathways are known to play an important role in recycling metabolically expensive guanine nucleobases and maintaining purine homeostasis. Although the *hpt1*Δ salvage deficient mutant showed no phenotype on rich or minimal media, we hypothesized that as the infection process might pose a greater metabolic strain, this enzymatic function may play an important role. We cultured *Caenorhabditis elegans*, a natural predator of *Cryptococcus*, on the wild-type, *hpt1*Δ and *hpt1*Δ *HPT1* strains. No significant difference was observed between survival times of the worms on the three strains on either rich or minimal media (Figure 3A). The addition of guanine to both the minimal and rich media again produced no significant difference between the three strains (data not shown), indicating that Hpt1-dependent salvage of purines is dispensable in the nematode model. We also tested the impact of loss of *HPT1* on virulence in the murine inhalation model of cryptococcosis. Again, there was no significant difference between the three strains (Figure 3B). Despite the extra stresses of increased temperature and the murine immune system, *de novo* purine biosynthesis alone proved sufficient for full virulence of *Cryptococcus* in a mammalian host.

### *De novo* GTP biosynthesis is critical to cryptococcal virulence *in vivo*

Metabolic enzymes in *Cryptococcus* have been shown previously to play ‘moonlighting’ roles in virulence, most famously urease [32], [33], and some are found in extracellular vesicles of virulence components secreted by *Cryptococcus*, including IMPDH [34], [35]. In the *C. elegans* virulence model on standard worm assay rich media or rich media supplemented with exogenous guanine, the *imd1*Δ strain did not display any significant difference in killing from the wild-type strain H99 or the complemented strain *imd1*Δ *IMD1* (Figure 3C and data not shown). On minimal media lacking guanine however, the *imd1*Δ strain was severely compromised for virulence (*p*<0.0001) in the worm.

**Figure 4:**
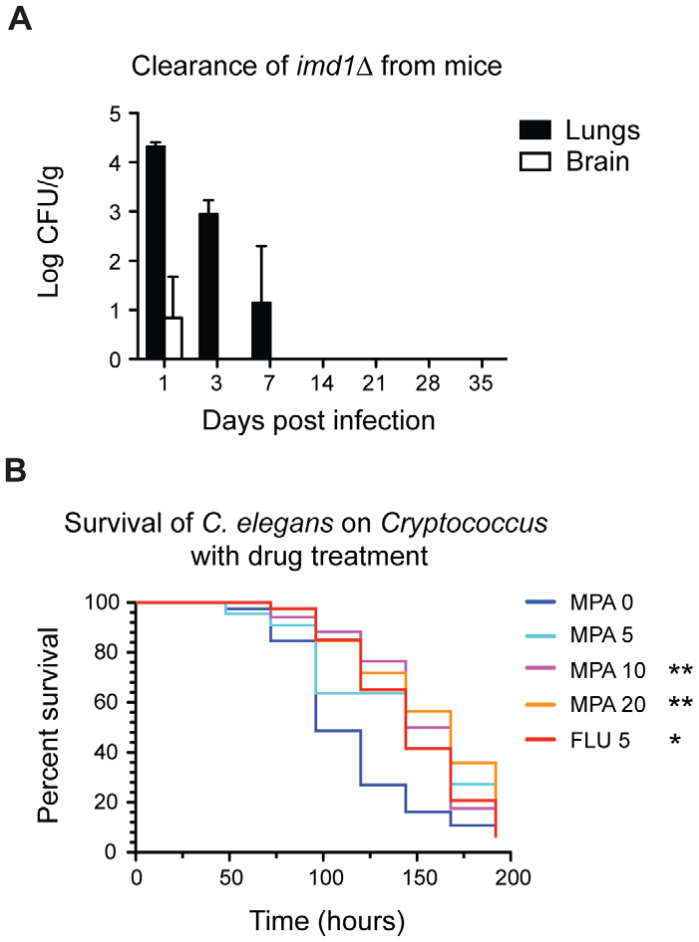
IMPDH is essential for pathogenicity in murine and nematode hosts. (A) Fungal burden of *imd1*Δ cells recovered from the lungs and brain of BALB/c mice infected with 5×10^5^ cells *via* nasal inhalation. Bars represent mean colony-forming units (CFU)/g ± standard error. (B) N2 Bristol young adult nematodes were co-cultured with *C. neoformans* wild-type strain H99 on minimal medium supplemented with 5, 10 or 20 µg/mL MPA or 5 µg/mL fluconazole. MPA significantly enhanced nematode survival over eight days, as did fluconazole treatment; *p*<0.05 *; *p*<0.01 **.

We next assessed the requirement for the GTP *de novo* pathway using the murine inhalation model of cryptococcosis. As in the nematode minimal media model, the *imd1*Δ mutant was unable to kill mice, even after 50 days (*p*<0.0001; Figure 3D). Certain functions of IMPDH are therefore critical for successful infection of a mammalian host by *Cryptococcus*. To more precisely examine the effect of loss of IMPDH on *Cryptococcus*, we determined the fungal burden for the *imd1*Δ strain for various organs at multiple timepoints during murine infection. Compared to the proliferation and dissemination that occurs during a wild-type infection, *imd1*Δ cell counts dropped rapidly after initial inoculation and the strain was not found in the lungs after just seven days (Figure 4A). Cells were found in the brain on day one (CFU = 328/g), although after three days these were no longer present. No dissemination to the spleen, liver or kidneys was observed (data not shown). It is unusual for cells to be recovered from the brain at 3 days after intranasal infection, which typically take at least 7 days to disseminate to the CNS from the lungs [36]. By comparison, at 14 days post infection when we observed total clearance, the wild-type strain is expected to have a log CFU/g of ∼8 in the lungs and ∼5 in the brain [37].

### IMPDH inhibitors protect *C. elegans* from infection in the nematode model of cryptococcosis

To validate IMPDH as an antifungal chemotherapeutic target *in vivo*, we utilized the *C. elegans* model of cryptococcosis to test the effect of IMPDH inhibitors on survival times. Nematodes were cultured on *Cryptococcus* in the presence of three concentrations of MPA in the media (5, 10 and 20 µg/mL) under typical assay conditions. As a control we simultaneously cultured nematodes in the presence of the fungistatic antifungal fluconazole (5 µg/mL). *C. elegans* cultured on *Cryptococcus* plus fluconazole displayed significantly enhanced survival (*p*<0.01). The addition of 10 or 20 µg/mL MPA also significantly enhanced survival times of worms (*p*<0.01; *p*<0.01) (Figure 4B). MPA is toxic to *C. elegans* with a LD_50_ of ∼90±10 µM (28.8 µg/mL) [38], however, the lower concentrations of MPA utilized appear tolerable and protective against *C. neoformans* infection; similar effects have been demonstrated with fluconazole, which is toxic to the worm at concentrations of ∼100 µg/mL but is protective against *C. albicans* infection at up to 32 µg/mL [39]. Control experiments did not reveal significant changes in mortality in worms cultured on 20 µg/mL MPA plus a food source *versus* worms on a food source alone (Figure S3).

### With the exception of a rare subtype, *Cryptococcus* is sensitive to the IMPDH inhibitor MPA

**Figure 5:**
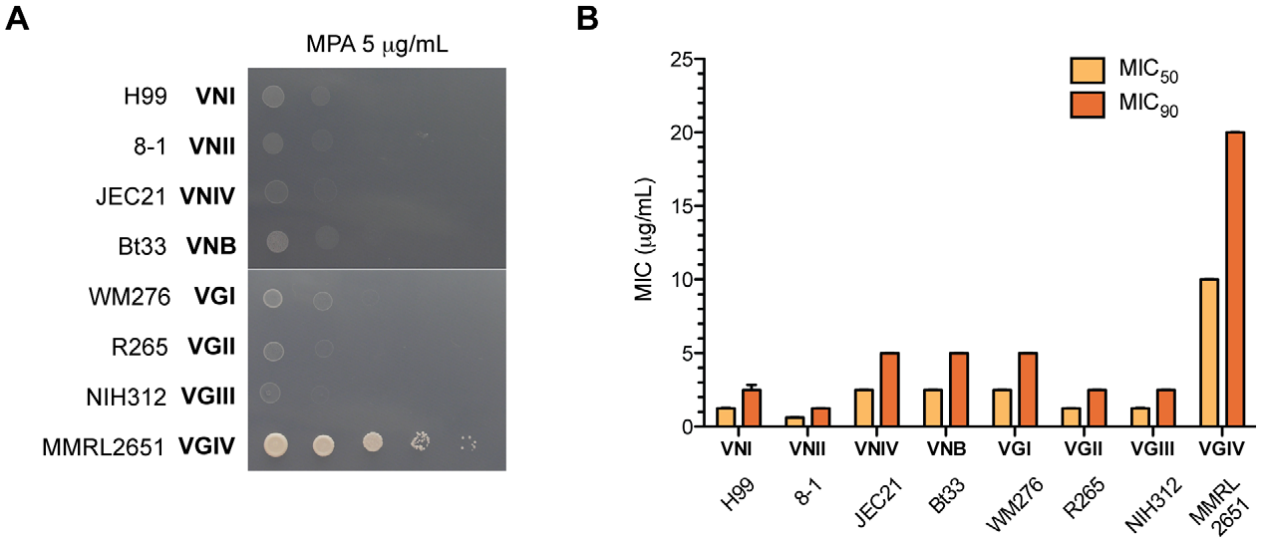
The *C. gattii* VGIV molecular type is resistant to MPA. (A) Resistance to the IMPDH inhibitor MPA was assessed in all haploid molecular types of the pathogenic *C. neoformans/C. gattii* species complex using a serial dilution spotting assay on YNB plus 5 µg/mL MPA. (B) Precise sensitivity to MPA was determined *via* MIC broth microdilution assay. MIC_50_ and MIC_90_ are the concentrations at which growth was inhibited by 50% or 90%, respectively. Standard error bars are displayed for all strains.

Based on our virulence studies, Imd1 but not Hpt1 could serve as a potential antifungal target. To test this hypothesis, the sensitivity of the entire *Cryptococcus* species complex to two IMPDH inhibitors, MPA and mizoribine, was examined. The *C. neoformans*/*C. gattii* species complex consists of eight haploid molecular subtypes, VNI, VNII, VNIII and VNB of *C. neoformans* and VGI, VGII, VGIII and VGIV of *C. gattii*, which vary in their distribution, ecology and epidemiology [40], [41]. While other fungal pathogens such as *C. albicans* are sensitive to mizoribine inhibition at very low concentrations, we did not observe any impact on *Cryptococcus*, even when high concentrations were used (100 µg/mL) in both MIC and serial dilution plate assays (data not shown) [21]. All subtypes were highly sensitive to low concentrations of MPA (5 µg/mL) in defined minimal medium, with one exception: the rare *C. gattii* VGIV molecular type (Figure 5A). Broth microdilution susceptibility assays supported this finding (Figure 5B). To confirm this observation, we tested a further 106 isolates and again found that the eight VGIV isolates in this collection were easily identified by their MPA resistance, while all others were sensitive (data not shown). VGIV MPA resistance was uniform and 100% reproducible, supporting this as an inherent characteristic of the VGIV molecular type.

**Figure 6:**
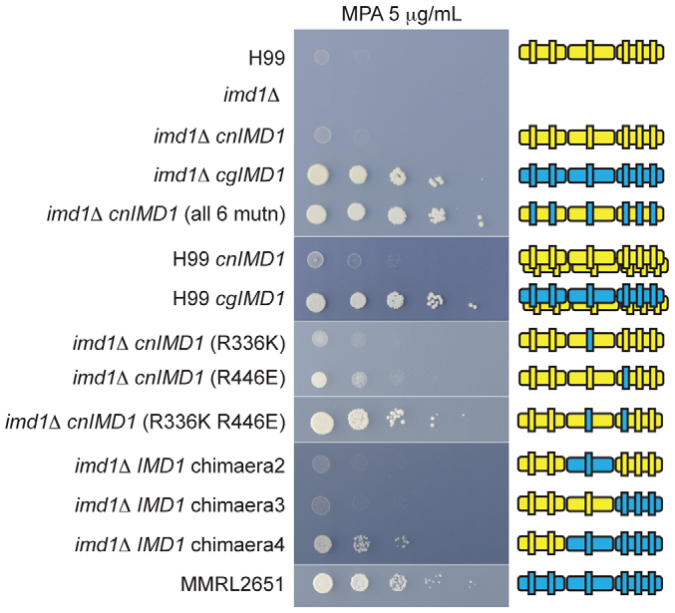
*C. gattii* IMPDH confers resistance to MPA, mediated by two amino acids. Serial dilution spotting assays of selected IMPDH mutants on YNB plus 5 µg/mL MPA. Robust growth on MPA is observed in the deletion mutant *imd1*Δ transformed with either the *CgIMD1* allele or any *IMD1* allele uniting the residues K336 and E446. Two copies of the MPA-sensitive *CnIMD1* allele (in H99 *CnIMD1*) result in only a very modest increase in resistance to MPA and IMPDH heterotetramerization (in H99 *CgIMD1*) does not appear to produce novel phenotypes. The juxtaposed image depicts which portions of the IMPDH allele are present, with yellow representing *CnIMD1* and blue representing *CgIMD1*, split into three thirds with the vertical bars depicting the six unique residues. Two proteins are depicted for the H99 background where the wild-type allele is also present.

We reasoned that resistance to MPA could arise from mutations in the *IMD1* ORF, overexpression of Imd1, increased *IMD1* copy number, or changes in drug uptake or efflux from the cell. To determine if MPA resistance could result from changes in expression level of IMPDH in *C. gattii* VGIV, we assessed the transcriptional response of *C. neoformans* (strain H99) and *C. gattii* (strain MMRL2651) *IMD1* to challenge by guanine and MPA *via* qRT-PCR. After treatment with guanine, both *C. neoformans* and *C. gattii IMD1* were significantly downregulated, consistent with *de novo* GTP synthesis not being required when abundant salvageable guanine is present (Figure S4). Importantly, no real difference was observed between the two strains' responses to either substrate or inhibitor, suggesting that resistance to MPA may in fact be an intrinsic property of the VGIV IMPDH isoform. To determine if either copy number or a feature of the *C. gattii* VGIV *IMD1* allele (*CgIMD1*) confer MPA resistance, we introduced both the *C. neoformans* var. *grubii* VNI allele (*CnIMD1*) from the MPA-sensitive strain H99 and *CgIMD1* from the MPA-resistant strain MMRL2651 into wild-type H99 and the *imd1*Δ mutant. In the wild-type H99 background, introduction of a second copy of *CnIMD1* resulted in only a very modest increase in MPA resistance, while introduction of the *CgIMD1* allele was sufficient to enable robust growth and VGIV-level resistance to MPA (Figure 6). In the *imd1*Δ deletion background, introduction of *CnIMD1* restores wild-type MPA sensitivity, while single-copy *CgIMD1* successfully confers VGIV-level MPA resistance (Figure 6).

### Spontaneous resistance to MPA does not arise readily in *Cryptococcus*

To present an attractive target for antifungal chemotherapy, spontaneous resistance to a drug should not arise readily through mechanisms such as single point mutations or rapid amplifications of target genes. In *C. albicans*, MPA-resistant strains were selected by extended incubation on sub-lethal concentrations of the drug resulting from a point mutation that changed the conformational equilibrium of IMPDH [21]. We performed equivalent selection using the VNI strain H99, but despite repeated attempts, we were unable to isolate colonies that were stably resistant to the drug after transfer onto media containing the same or increasing concentrations of MPA (data not shown).

### *Cryptococcus gattii* VGIV IMPDH has multiple unique polymorphisms

To investigate the nature of the VGIV resistance to MPA, we compared the *IMD1* coding region between all known haploid molecular types. While there are 22 residue differences between VNI (MPA-sensitive) and VGIV (MPA-resistant) IMPDH, there are only six residues unique to the VGIV subtype: three conservative and three nonconservative substitutions (Figure S5). To establish the precise mechanism for MPA resistance, we introduced the six VGIV unique substitutions into the sensitive VNI enzyme. No single mutation was sufficient to confer VGIV-level resistance to MPA when introduced into the *imd1*Δ or the wild-type background, however the simultaneous introduction of all six mutations into the *CnIMD1* allele did confer MPA resistance (Figure 6 and Figure S6). This finding confirms that a combination of these six residues is sufficient to confer MPA resistance, which explains our inability to isolate spontaneous MPA-resistant isolates of VNI. We therefore generated VNI/VGIV IMPDH chimeras using convenient restriction sites that split the IMPDH gene into three parts to combine *CnIMD1* and *CgIMD1* into six possible configurations. Only the construct comprising the first third of *CnIMD1* and the last two-thirds of *CgIMD1* (bearing R446E, G450A and A500V) conferred VGIV-level resistance to MPA (Figure 6 and Figure S6). Based on this observation we employed site-directed mutagenesis of the MPA sensitive VNI allele to introduce the conservative R336K substitution (the only unique residue in the middle fragment) in turn with each of the three residues from the last fragment; the combination of K336 and E446 was sufficient to confer MPA resistance (Figure 6 and Figure S6). Again, transformation of these constructs into the wild-type H99 background yielded levels of MPA resistance comparable with the *imd1*Δ deletion strain, suggesting that simple gene amplification may be insufficient to produce robust resistance, and the resulting mixed IMPDH heterotetramers in these strains did not appear to possess novel drug-resistant characteristics.

### Steady-state kinetics of CnIMPDH and CgIMPDH

The catalytic mechanism of IMPDH involves a transition from a dehydrogenase to a hydrolase reaction; initially, the substrate IMP and cofactor NAD^+^ bind, IMP is then oxidized and forms a covalent transition state complex *E*-XMP* *via* a catalytic cysteine, while NAD^+^ is simultaneously reduced to NADH. The enzyme then undergoes a conformational change whereby a large mobile flap folds into the vacant NAD^+^ site, carrying a catalytic arginine residue that activates a water molecule to hydrolyze the *E*-XMP* covalent complex, releasing XMP [26], [42], [43]. To investigate if resistance to MPA is due to functional differences between CnImd1 and CgImd1, we determined the steady-state kinetic parameters (Figure S7). Plots of velocity *versus* IMP concentrations were best fit by the Michaelis-Menten equation (*K_m_*_(IMP)_ = 84±12 µM, *K_m_*_(NAD)_ = 634±97 µM for CnImd1 and *K_m_*_(IMP)_ = 178±15 µM, *K_m_*_(NAD)_ = 532±65 µM for CgImd1). However, both enzymes showed strong NAD^+^ substrate inhibition above concentrations of 1,500 µM (*K_ii_*_(NAD)_ = 4,400±900 µM for CnImd1 and *K_ii_*_(NAD)_ = 4,000±600 µM for CgImd1), which is common for IMPDH and is attributed to the formation of an *E*-XMP*•NAD^+^ complex but meant that the data had to be fitted to a modified version of the Michaelis-Menten equation that takes substrate inhibition into account. The *K_m_* values for both enzymes were significantly different from the values for both human isoforms of IMPDH (Table 1) while the turnover numbers were similar (*k*_cat_ = 1.5±0.1 s^−1^ for CnImd1 and *k*_cat_ = 2.2±0.2 s^−1^ for CgImd1); IMPDH has a generally low turnover and the rate for *Cryptococcus* is similar to those determined for IMPDHs from humans, other fungi and parasites [19], [21], [28].

**Table 1:** Kinetic parameters for IMPDH from characterized species.

IMPDH	*k*_cat_ (s^−1^)	*K_m_*_(IMP)_ (µM)	*K_m_*_(NAD)_ (µM)	*K_ii_*_(NAD)_ (mM)	*K_ii_*_(MPA)_ (nM) *vs.* NAD^+^	*K_ii_*_(MPA)_ (nM) *vs.* IMP
*E. coli*	13.0	61	2000	2.8	≥10000	ND
*C. parvum*	3.3	29	150	2.9	9300	ND
*T. foetus*	1.9	1.7	150	6.8	9	ND
*H. sapiens* type I	1.8	14	42	ND	11	ND
*H. sapiens* type II	0.4	4	6	0.6	6	ND
*A. nidulans*	0.7	10	170	1.5	25 (UC)	22 (UC)
*P. chrysogenum* A	0.8	40	290	2.4	ND	ND
*P. chrysogenum* B	0.0075	600	640	NA	ND	ND
*P. brevicompactum* A	0.7	130	340	4.7	500 (UC)	450 (NC/mixed)
*P. brevicompactum* B	0.4	1400	790	NA	14000 (UC)	20000 (NC/mixed)
*C. albicans*	6	60	3500	1.5	11	ND
*C. neoformans* VNI	1.5±0.1	84±12	634±97	4.4±0.9	240±18 (UC)	198±63 (NC/mixed)
*C. gattii* VGIV	2.2±0.2	178±15	532±65	4.0±0.6	96±5 (UC)	107±31 (NC/mixed)

Steady-state and inhibition constants for species with well-characterized IMPDHs. UC, uncompetitive; NC, noncompetitive; ND, no data; NA, not applicable. Values are from [19], [21], [28], [58].

### Inhibitor kinetics of CnIMPDH and CgIMPDH

MPA is an NAD^+^ analogue and an uncompetitive inhibitor that traps the *E*-XMP* enzyme-substrate covalent complex by competing with the flap for the vacant NAD^+^ site [44]. To further investigate the mechanism behind increased MPA resistance, we examined the response of both enzymes in the presence of inhibitor and varying amounts of both substrates. The concentration of MPA used was similar to the concentration of enzyme in the assays, which necessitated tight-binding inhibitor treatment, as found in *C. albicans* and *Aspergillus nidulans* IMPDH [45]. MPA dependent changes in kinetic parameters were studied for both isoforms and were best described by an uncompetitive, tight binding inhibition mechanism *versus* NAD^+^, indicative of formation of *E*-XMP*•MPA complexes, as has been observed for multiple organisms (Figure S8). The *K_ii_* for the MPA-sensitive CnIMPDH allele (240±18 nM) is higher than the *K_ii_* observed for the well-characterized human type I, human type II and *C. albicans* IMPDHs, suggesting that the NAD^+^ site (Table 1), which is also the drug-binding site, is functionally different in the wild-type *Cryptococcus* enzyme. Curiously, the inhibition constant for MPA is actually lower for the MPA-resistant CgImd1 enzyme (*K_ii_* = 96±5 nM), although it displays a greater *k*_cat_/*K_m_*_ (NAD)_ ratio (4,135±63 M^−1^ s^−1^) compared to CnImd1 (2,366±39 M^−1^ s^−1^), suggesting greater enzymatic efficiency. We note that fits for noncompetitive/mixed tight-binding inhibition for the MPA-resistant CgImd1 enzyme were extremely similar to those obtained for uncompetitive tight-binding inhibition and yield a much higher *K_ii_* (293±76 nM; data not shown), which may indicate some affinity of the inhibitor for other enzyme forms. Surprisingly, fits of MPA *versus* IMP for both enzymes were best described by noncompetitive/mixed patterns of inhibition (Figure S8), with a *K_ii_* = 198±63 nM for CnImd1 and a *K_ii_* = 107±31 nM for CgImd1. This suggests that in *Cryptococcus* IMPDH, MPA binds to additional enzyme forms such as free enzyme or *E*-IMP. Such a scenario is also found in MPA-resistant *Penicillium brevicompactum* IMPDH, where the initial hydride transfer step (as opposed to the subsequent hydrolysis step) is either partially or completely rate-limiting, resulting in the predominate enzyme form being *E*-IMP-NAD^+^ instead of *E*-XMP*, conferring MPA resistance [46].

### Crystal structure of CnImd1

**Figure 7:**
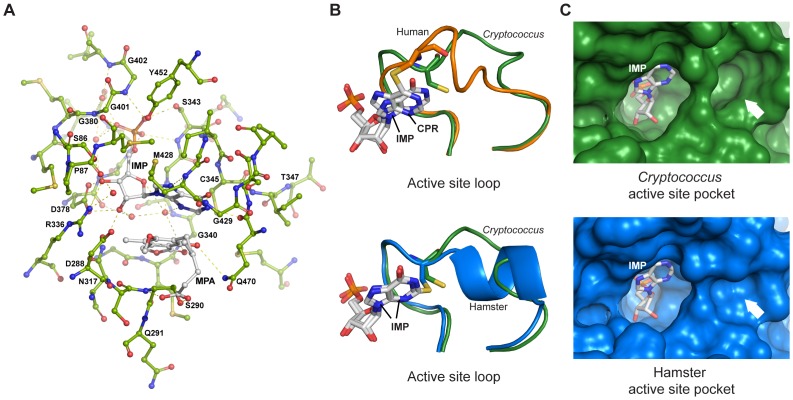
Structure of *C. neoformans* IMPDH. (A) Active site residues of *C. neoformans* IMPDH with IMP and MPA ligands in ball-and-stick representation, with hydrogen-bonding interactions indicated by dashed lines. Figure displays residues within 4 Å of the active site except for T347, which adopts a strikingly different conformation in the *Cryptococcus* enzyme. (B) Residues 339–356 of *Cryptococcus* IMPDH (green) form the active site loop and are highly conserved. Comparison of the *C. neoformans E*•IMP•MPA complex active site loop conformation with the hamster *E*-XMP*•MPA complex and the human type I IMPDH in complex with the substrate analogue 6-chloropurine ribotide (CPR) are shown. IMP, CPR and the catalytic cysteine are shown in stick representation, while MPA is omitted. (C) Surface representation of the active site pocket of *C. neoformans* and Chinese hamster IMPDH. The white arrow indicates the region around T347, which forms a unique, potentially exploitable cavity in the fungal enzyme. MPA is omitted for clarity.

To provide a foundation for fungus-specific inhibitor development, we determined the crystal structure of the clinically relevant *C. neoformans* var. *grubii* IMPDH in complex with the substrate IMP and the inhibitor MPA. CnImd1 forms a tetramer and in the final model each monomer contains 395 of the 544 residues in the typical eight-stranded α/β barrel structure, plus one molecule each of IMP and MPA bound in the active site, consistent with our functional observations suggesting an uncompetitive mechanism of enzyme inhibition (Figure 7A). The large accessory subdomain between residues 130–244 and residues 438–449 from the mobile flap have no interpretable electron density, common to previous structural studies on this class of enzymes.

Many of the active site residues are invariant, and the purine ring, the ribose group and the nucleotide phosphate group of IMP make mostly conserved interactions to those found in the Chinese hamster IMPDH:IMP:MPA structure [26]. MPA makes far fewer interactions in the cryptococcal IMPDH:IMP:MPA complex than observed in the hamster IMPDH structure, perhaps accounting for some of the differences in *K_i_* between the mammalian and fungal enzyme. In particular, T347 (*Cryptococcus* numbering) faces away from the active site in the *Cryptococcus* enzyme and does not bind MPA, while several other residues including N317, D288 and G340 participate in fewer interactions with the ligands in the cryptococcal complex. The six unique residues found in CgImd1 map to the amino terminal region near a monomer-monomer interface (V55M), the accessory cystathionine-β-synthase (CBS) domain (A153T), adjacent to the active site loop in the NAD^+^ and MPA-binding cavity (R336K), close to a catalytic arginine in the movable flap (R446E and G450A), and near the carboxy terminus (A500V). R/K336 forms hydrogen bonds with IMP and MPA in the hamster enzyme, and R/E446 and G/A450 are near the catalytic arginine (R458) that resides on the mobile flap. Of note, one of the two residues responsible for MPA resistance in *C. gattii* VGIV IMPDH, R336 (K336 in the resistant enzyme), forms direct contacts with only IMP in the cryptococcal complex, suggesting a possible mechanistic explanation for the failure of the R336K single mutant to exhibit any altered sensitivity to MPA. Substitution of R336 with K336 *in silico* increases the distance from the amine nitrogen of R336K to the IMP ribose O3 hydroxyl from 3.1 Å to 3.9 Å, which would lead to the loss of contacts with IMP.

Comparison of the hamster and cryptococcal IMPDH active site loops with IMP and MPA-bound states indicates differences in conformations, despite considerable similarity at sequence level; in the human type I IMPDH structure (bound to the IMP analog 6-chloropurine ribotide) this loop adopts yet another distinct conformation (Figure 7B). The change in orientation of the loop in CnImd1 creates a large, *Cryptococcus*-specific pocket in the active site (Figure 7C). There is considerable flexibility in the conformation of the active site loop observed in structures of different enzyme complexes, which suggests that it can mimic the changes during the reaction cycle where it may modulate closure of the flap [47]. While IMP is covalently bound to the catalytic cysteine in the hamster and human structures, which may be responsible for the altered orientation, comparison with *Tritrichomonas foetus* IMPDH bound to IMP or mizoribine phosphate (where the ligands are not covalently bound in either structure) suggests the conformation observed in the cryptococcal complex to be unique, thus offering a potential avenue for species-specific inhibition. IMPDH crystal structures have been demonstrated to provide a reliable indication of the position of the loop at different parts of the reaction cycle, suggesting the pocket observed in the *Cryptococcus* structure to be a legitimate target [47].

**Figure 8:**
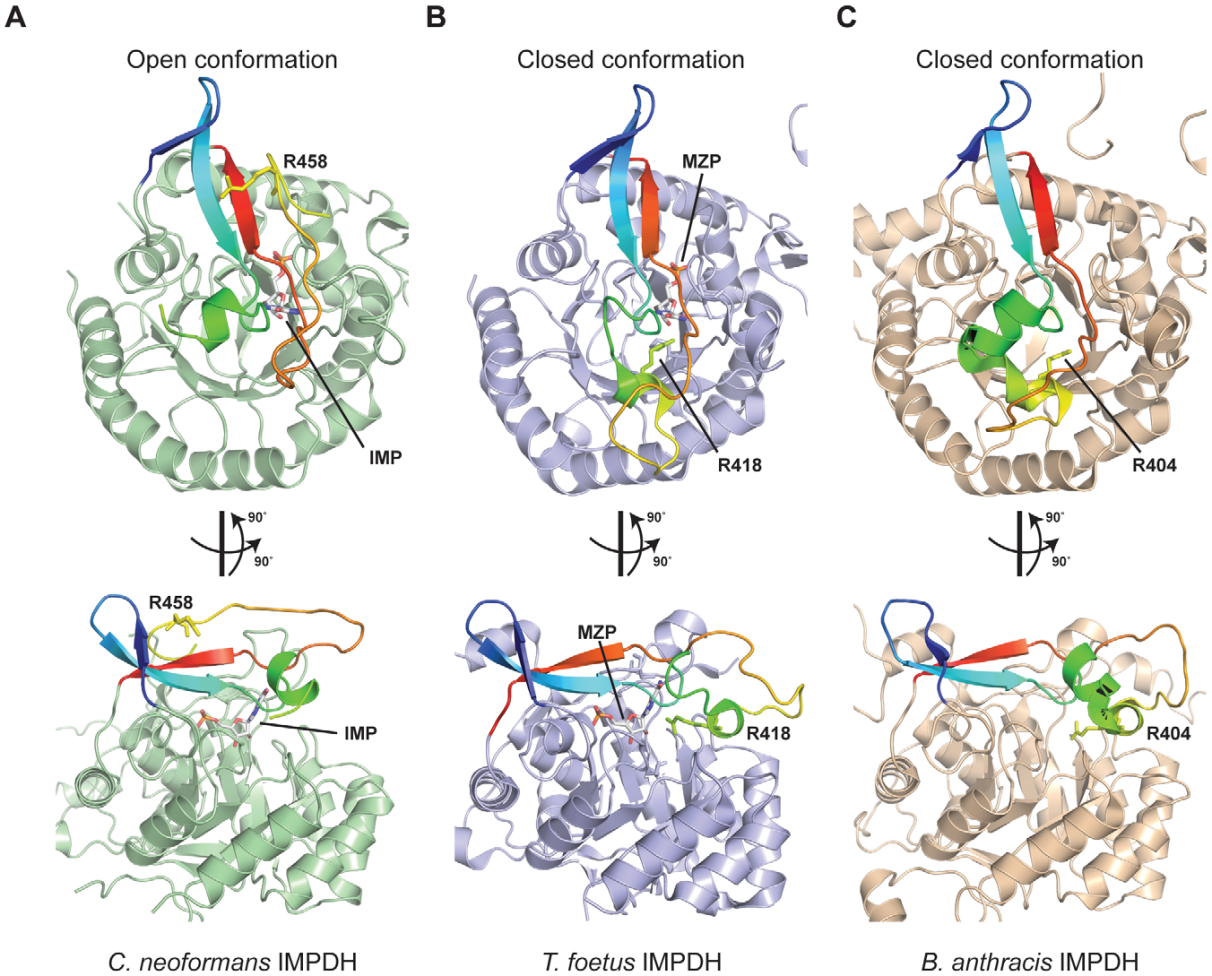
The open and closed conformations of the IMPDH mobile flap. (A) The *Cryptococcus neoformans* IMPDH structure indicates that the flap adopts an open conformation in the ligand-bound state, with the catalytic R458 folded above the active site. The flap is rainbow colored and the catalytic arginine and IMP in the active site are depicted in stick representation. (B) The *Tritrichomonas foetus* IMPDH structure in complex with mizoribine phosphate (MZP) is in the closed conformation, with the flap extending out over the active site pocket and the catalytic arginine positioned underneath. (C) The *Bacillus anthracis* structure shows the unbound apoenzyme also adopts the closed conformation, with the flap closed over the active site.

The cryptococcal IMPDH structure is unique in having most of the mobile flap, except for 12 residues that encompass a small insertion region unique to *Cryptococcus*, sufficiently ordered to permit all but three residues corresponding to the flap in human IMPDH to be observed. The only comparable structural evidence for the flap is presented for the parasite *T. foetus* IMPDH complexed with mizoribine phosphate, in which the flap is in the closed conformation within the cofactor site [29] and the *Bacillus anthracis* apoenzyme structure [48]. The cryptococcal structure reveals the flap in the ‘open’ conformation, as would be required for the initial dehydrogenation before the flap folds into the NAD^+^ site to hydrolyze the covalent enzyme intermediate. The flap is folded back onto itself, with residues A455–A465 facing the following V466–V476, with the catalytic R458 positioned more than 13 Å away from the C2 carbon of the IMP purine ring (Figure 8A). Comparison with both the *T. foetus* and *B. anthracis* enzymes suggests that the flap undergoes significant conformational rearrangement, swinging completely around and underneath itself to bring the catalytic arginine into close juxtaposition to the substrate (Figure 8B). Despite being uncomplexed with substrate or inhibitor, the *B. anthracis* structure also clearly shows the enzyme in the closed conformation, with the flap folded into the active site (Figure 8C). Superposition of the bacterial enzyme onto the fungal enzyme reveals that the mobile portions of the flap comprise residues H436-A465 (30 residues in *Cryptococcus* but only 15 residues in human), while the remaining residues of the flap, Y414-E435 and V466-K479, appear stationary. Comparison of the open conformation of the *C. neoformans* enzyme (MPA-sensitive) with the closed conformation of the *T. foetus* and *B. anthracis* enzymes (both MPA-resistant) readily demonstrates how MPA can more easily access the cofactor site in enzymes where the conformational equilibrium favors the open state [44]. Unfortunately the second residue responsible for MPA resistance in *C. gattii* VGIV, E446 (R446 in the sensitive enzyme), was not observed in the electron density, however this is a strong indicator of considerable flexibility within the immediate structural context of this residue, indicating it unlikely to be proximal to the well-ordered active site in the open conformation. However, as the flap undergoes structural rearrangement, the residue is likely to be brought into closer proximity to the active site pocket during the reaction cycle.

## Discussion

New drug targets and novel approaches to treatment are urgently needed as the immunocompromised population grows and the burden of opportunistic fungal infections increases. We have examined and characterized two of the key enzymes from the GTP *de novo* biosynthetic and salvage pathways, revealing the potential of this pathway as a target of anticryptococcal therapy. We discovered that only the rare *C. gattii* VGIV molecular type is resistant to a natural product inhibitor of IMPDH, presenting an opportunity to investigate the evolution of drug resistance in IMPDH in the *Cryptococcus* species complex, and whether this poses a risk to potential therapies.

GTP synthesis appears to be a natural antimicrobial target, as both MPA and mizoribine are produced as secondary metabolites by various mould species. The ubiquity of MPA resistance among *C. gattii* VGIV isolates suggests that it may be an adaptation, perhaps to competitors in its environmental niche. Very little is known on the ecology of VGIV isolates in particular, and while it has been isolated from clinical samples from a small number of countries in South America, sub-Saharan Africa and the Indian subcontinent, reports of environmental isolates are limited to certain trees (primarily the Indian almond tree *Terminalia catappa*) from Colombia and Puerto Rico [49]. The VGIV subtype is nevertheless extremely rare, while the clinically-relevant VNI type is widespread and exquisitely sensitive to MPA.

The finding that the purine salvage deficient *hpt1Δ* mutant was fully virulent in both the worm and mouse model was somewhat surprising, given the intense nutritional and metabolic stresses and hostile immune system pressures *Cryptococcus* is under during the infection process. Similar PRTase mutants in the apicomplexan parasites *Leishmani donovani* and *Toxoplasma gondii* are viable and virulent however, although many display certain fitness defects or partial attenuation of infectivity [50], [51]. Given that *de novo* purine biosynthesis is an energy intensive process, salvage of purines from a host could serve as an excellent way to conserve energy during infection; as the *hpt1*Δ mutant is not perturbed for virulence, this may reflect a generally low concentration of salvageable purines during the infection process. This model is supported by the avirulence of the *imd1Δ* mutant; generating a ready supply of GTP *via* the *de novo* pathway is obviously of critical importance to the fungus during infection.

These findings are consistent with other studies demonstrating that IMPDH and *de novo* GTP biosynthesis mutants from a range of pathogenic bacteria are attenuated for virulence [52], and in pathogenic *E. coli*, *Salmonella enterica* and *B. anthracis*, *de novo* purine biosynthesis has been proposed to be the single most critical metabolic function for growth in human serum and blood [53]. Purine *de novo* biosynthesis may potentially be rate-limiting to growth of *Cryptococcus* during infection, and therefore present an outstanding target for anti-cryptococcal chemotherapy. Furthermore, in contrast to the simple guanine auxotrophy of *S. cerevisiae* and *E. coli* IMPDH mutants [54], [55], the *imd1Δ* mutant of *Cryptococcus* exhibits a range of pleiotropic phenotypic defects even when supplemented with exogenous guanine, including slow growth, attenuated virulence factor expression, and impaired dissemination and avirulence in two animal models of infection. Intriguingly, recent studies have revealed that both IMP dehydrogenase and GMP synthase, the first and second enzymes of *de novo* GTP biosynthesis, act as transcriptional repressors in *Drosophila* [35], [56], while the accessory CBS domain of IMPDH has been demonstrated to regulate purine homeostasis in *E. coli* [54], [57]. It would be valuable to investigate the potential regulatory roles of IMPDH in *Cryptococcus* and establish any contributions to pathogenicity.

That IMPDH is absolutely critical for virulence of *Cryptococcus*, and the observation that the most clinically prevalent forms of *Cryptococcus* are exquisitely sensitive to the IMPDH-inhibiting drug MPA, is encouraging for the investigation of IMPDH and *de novo* GTP biosynthesis as an antifungal drug target. Kohler *et al.* [21] previously established that the *C. albicans* form of the enzyme displays an altered kinetic profile relative to human IMPDH, suggesting that fungus-specific inhibitors could be developed that target the MPA binding site. Functionally, the two *Cryptococcus* enzymes appear quite similar to other fungal IMPDHs, including the *C. albicans* and *A. nidulans* enzymes, and GTP biosynthesis is critical for virulence of both *C. albicans* and *A. fumigatus* (closely related to *A. nidulans*). Cryptococcal IMPDH also shows significant differences from the human enzyme, despite ∼56% sequence identity. The *K_m_* for both IMP and the cofactor NAD^+^ are substantially different from the human forms, suggesting that there are exploitable aspects of the reaction cycle of the cryptococcal enzyme. It is less clear why VGIV CgImd1 should have a lower *K_ii_* for MPA than the VNI variant. Residues K336 and E446 together appear to play a pivotal role in *C. gattii* VGIV resistance, as demonstrated by their introduction into the sensitive form, perhaps by altering the affinity of the flap for the cofactor site or other changes in the enzyme mechanism or conformation. The dynamics of the flap movement are known to be key to resistance to cofactor mimic drugs in IMPDH, and key mutations can alter the preferred state from open and MPA-sensitive (the state observed in our structure, with the flap poised above the active site and the cofactor site exposed), to closed, which restricts access of MPA to the cofactor site and confers resistance [44]. Residues close to the catalytic arginine are involved in hydrogen bonding interactions when the flap folds into the NAD^+^ site in *T. foetus* and presumably other species [29]. The combination of residues observed in *C. gattii* VGIV is reminiscent of the well-characterized MPA-resistant *T. foetus* enzyme, which requires K310 (equivalent to *Cryptococcus* K336) and E431 (equivalent to *Cryptococcus* Q470, also on the flap) in tandem to provide robust resistance [30]. Our data suggests that while MPA affinity increased, a compensatory increase in enzyme catalytic efficiency may have occurred through the combination of K336 and E446. Recent work also demonstrates that if the initial hydride transfer step becomes rate-limiting, this reduces accumulation of the enzyme-substrate covalent complex and thus reduces susceptibility to MPA inhibition. Notably, this has occurred in MPA-resistant *Penicillium brevicompactum* IMPDH without significant residue changes around the substrate/cofactor binding sites [46], [58]. Our work demonstrates that MPA binds to additional cryptococcal IMPDH forms (an uncompetitive inhibitor should bind only to the enzyme-substrate covalent complex), suggesting the enzyme to be fundamentally functionally different from the human isoforms.

To provide a solid foundation for chemotherapeutic agent development, we have determined the structure of *C. neoformans* var. *grubii* IMPDH in complex with IMP and MPA. While the accessory domain is presumably disordered and was not observed, we were able to successfully model the residues comprising the mobile flap domain, which to date had only been structurally characterized in the closed conformation in the *T. foetus* and the *B. anthracis* enzymes [29], [48]. While *Cryptococcus* IMPDH possesses an insertion within the flap that was mostly disordered and there is considerable flexibility associated with the flap region, the structural evidence suggests that the flap undergoes a drastic conformational change during the reaction cycle, which has been demonstrated functionally but not structurally in previous studies [27], [59], [60]. In the open enzyme conformation, the catalytic arginine is positioned away from the active site, with the flap undergoing a 270° swinging rotation to perform the hydrolysis reaction. The near-complete flap structure in the open orientation will aid future drug design efforts, as the flap folds into the cofactor site – a critical target site for inhibitors. The cofactor site, and the adenosine subsite in particular, also present an opportunity for fungus-specific inhibitor development, as there are a number of critical residues that vary in the *Cryptococcus* enzyme; for example, the adenine ring of NAD^+^ likely stacks between R267 and Y296 in *Cryptococcus* IMPDH, and these two residues differ from the corresponding H253 and F282 in the human enzyme. The eukaryotic parasite *T. foetus* shares these two substitutions, which have been suggested as potential species-specific targets [61]. The adenosine portion of the cofactor site is also a monomer-monomer interface, and one of the more variable parts of the enzyme between species. Recent strategies extend inhibitors from the nicotinamide subsite (where MPA binds) out to the adenosine subsite to increase specificity [44], [62]. The unique pocket in the active site loop region of *Cryptococcus* IMPDH will enable the design of inhibitors with moieties that occupy the cavity, which should be selective for the fungal enzyme over the human enzymes.

Overall, we have demonstrated that *de novo* GTP biosynthesis is crucial for cryptococcal virulence factor expression and pathogenesis, and multiple exploitable avenues exist in IMPDH to develop inhibitors that are specific for the fungal enzyme. This work lays the foundation for purine biosynthesis as a legitimate antifungal target, and the functional similarity between the *Cryptococcus* and *Candida* enzymes presents an intriguing opportunity to develop novel IMPDH inhibitors that could lead to effective therapy against a spectrum of fungal pathogens.

## Materials and Methods

### Ethics statement

The *Cryptococcus* murine virulence assay protocol was approved by the Molecular Biosciences Animal Ethics Committee of the University of Queensland (AEC# SCMB/473/09/UQ/NHMRC). Assays were conducted in accordance with the guidelines in the Australian code of practice for the care and use of animals for scientific purposes by the National Health and Medical Research Council. Infection and euthanasia were performed under methoxyfluorane anesthesia, and all efforts were made to minimize suffering *via* strict adherence to the Guidelines to promote the wellbeing of animals used for scientific purposes by the National Health and Medical Research Council.

### Strains and media

*C. neoformans* and *C. gattii* strains were cultured in YPD or YNB (Becton Dickinson, Franklin Lakes NJ) media at 30°C. *imd1Δ* mutants are guanine auxotrophs and were grown on minimal YNB media supplemented with 1 mM guanine for all manipulations; *ade2Δ* and *hpt1Δ ade2Δ* mutants are adenine auxotrophs and were maintained on rich media. Cloning and plasmid propagation was performed in *E. coli* strain Mach1 (Life Technologies, Carlsbad CA). Genomic DNA for *E. coli* gene isolation was prepared from strain K12. *C. elegans* strain N2 Bristol was maintained at 20°C on lawns of the *E. coli* uracil auxotroph strain OP50 on Nematode Growth Medium using standard procedures [63]. Recombinant *Cryptococcus* IMPDH was heterologously expressed in *E. coli* strain BL21(DE3)pLysS (Merck, Darmstadt, Germany). MPA, mizoribine, 5-fluoro-2′-deoxyuridine, G418, NAD^+^, Tris, Bis-Tris, glycine, NaCl, KCl, DTT, EDTA, DMSO, uric acid, inosine, hypoxanthine, xanthine, xanthosine, guanine, guanosine, adenine, adenosine, IMP, AMP, XMP and GMP were purchased from Sigma (St Louis, MO). Nourseothricin (clonNAT) was purchased from Werner BioAgents (Jena, Germany). MPA was dissolved in DMSO and was used at a concentration of 5 µg/mL. Supplemental purines when required were added to a final concentration of 1 mM [63].

### Molecular techniques

Standard molecular techniques were performed as described by Sambrook *et al.* [64]. *C. neoformans* genomic DNA for Southern blot analysis was prepared as described [65]. Oligonucleotides used are given in Table S1. For Southern hybridizations, DNA was digested with appropriate enzymes, electrophoresed on TAE-agarose gels and blotted onto Hybond-XL membrane (GE Healthcare, Little Chalfont, United Kingdom) using standard procedures. Probes were generated using the Rediprime II Random Prime Labeling Kit (GE Healthcare) with PCR products from H99 or MMRL2651 template DNA, and α^32^P dCTP (PerkinElmer, Waltham MA). Blots were hybridized overnight at 65°C and membranes were exposed onto Fuji Super RX medical X-ray film (Fujifilm, Tokyo, Japan). Splice overlap PCR, knockout transformations and gene complementations were performed as described [66]–[69], using particle delivery using a BioRad He-1000 Biolistic Device (Bio-Rad, Hercules CA).

### Bioinformatic analysis

The unpublished *C. neoformans* var. *grubii* genome is available from the Broad Institute of MIT and Harvard (http://www.broadinstitute.org/annotation/genome/cryptococcus_neoformans/MultiHome.html). To amplify genes from *C. gattii* strain MMRL2651, primers were designed from the closely related *C. gattii* strains WM276 and R265, which are also available from the Broad Institute (http://www.broadinstitute.org/annotation/genome/cryptococcus_neoformans_b/MultiHome.html). Purine biosynthetic genes were identified using reciprocal best-hit BLAST analysis and annotated using MacVector 9.5 (MacVector, Cary NC) [70]. Sequencing was performed at the Australian Genome Research Facility (Brisbane, Australia) and sequence traces were analyzed using Sequencher 4.7 (Gene Codes, Ann Arbor MI). Sequences for *Cryptococcus* IMPDH from strains 8-1 (VNII), Bt33 (VNB), NIH312 (VGIII) and MMRL2651 (VGIV) are available from GenBank under the accession numbers FJ418778–FJ418781.

### Phenotypic and virulence factor assays

For growth curve analysis, cells were cultured in RPMI 1640 media (Life Technologies) plus 2% glucose, 10% fetal bovine serum (Life Technologies) and 1 mM guanine. Cells were freshly cultured on YNB or YNB plus guanine plates, washed, diluted to an OD_600_ of 0.05 and grown in 50 mL cultures with shaking at 180 rpm at 30°C for a total of five days and monitored spectrophotometrically. Melanization assays were performed on solid or liquid *Cryptococcus* melanization media containing l-DOPA (l-3,4-dihydroxyphenylalanine) and 10 mM asparagine at 30°C and 37°C [71]. Spectrophotometric analysis was performed on culture supernatant at OD_475_ using a NanoDrop spectrophotometer [72]. Capsule growth was induced *via* overnight growth in RPMI 1640 media plus 2% glucose and 10% fetal bovine serum, and stained with India ink (Becton Dickinson) to visualize capsule. Capsule sizes were determined from phase contrast images using CellProfiler image analysis software [73]. An algorithm was developed to identify the cryptococcal capsule and cell wall boundaries by segmentation. Clumped cells, those outside size bounds and those touching the border of the image were excluded. Geometric parameters were measured from the identified capsule and cell wall boundaries, and the average of the major and minor axes from an ellipsoid fit used to estimate both capsule size and cellular diameter. Capsule diameter was expressed as a relative percentage of cell size. At least 10 independent images were used from each sample with at least 50 and typically 100–200 cells measured from each replicate; all measurements were performed in biological triplicate [74]. Mating tests were performed on Murashige-Skoog or V8 media plus 100 µg/mL *myo*-inositol (pH 5.0) using the *MAT* **a** H99 isogenic strain KN99**a**, as described [75], [76].

### Nematode virulence assays

Assessment of cryptococcal virulence in the *C. elegans* model system was performed as described [77]. *Cryptococcus* strains were grown on either rich (Brain-Heart Infusion media, Becton Dickinson) or minimal (Nematode Growth Medium plus *S. cerevisiae* Synthetic Complete Supplement Mixture) media with or without supplemental guanine. For *C. elegans* plus antifungal assays, 5, 10 or 20 µg/mL MPA or 5 µg/mL fluconazole were added to the minimal media plates. For the control drug assays, worms were cultured on NGM supplemented with 6 µM 5-fluoro-2′-deoxyuridine to prevent egg laying, plus either OP50 bacteria or heat-killed OP50 bacteria, with or without 20 µg/mL MPA.

### Murine virulence assays and organ burden analysis

Assessment of cryptococcal virulence in the murine model system was performed as described [78], with slight modification. Due to the propensity for the *imd1*Δ strain to clump in liquid media, all strains were freshly grown on YNB or YNB plus guanine plates overnight before washing and counting for infection. Kaplan-Meier survival curves were plotted using the software package GraphPad Prism 5.0 (GraphPad Software, San Diego CA), and statistical significance was assessed using a log-rank test, with a *p* value <0.05 considered significant. For organ burden analysis, brain, lungs, liver, spleen and kidneys were harvested from three mice per time point, weighed, homogenized and plated in serial dilution to determine colony-forming units per gram organ weight.

### Gene expression analysis

Quantitative reverse transcription PCR analysis was performed as described [79]. Overnight cultures of strains H99 and MMRL2651 were grown for 16 hours to mid-log phase, before addition of MPA (5 µg/mL) or guanine (1 mM). Cells were grown for 15 minutes, 60 minutes or 240 minutes before harvesting and flash freezing in liquid nitrogen. Total RNA was extracted using TriZOL reagent (Life Technologies) and cDNA was generated using Superscript III (Life Technologies).

### MIC determination and MPA resistance

MIC susceptibility testing for MPA was performed using the broth microdilution method according to CLSI (CLSI M27-A2) modified for *Cryptococcus neoformans* [80]. Strains tested are found in Table S2. For analysis of spontaneous MPA resistance, 1×10^10^ cells each of H99 overnight cultures were plated onto YNB with sub-lethal concentrations of MPA (1–5 µg/mL) as described [21]. Plates were incubated at 30°C for two months. Putatively resistant colonies were subcultured onto fresh YNB plus MPA plates.

### Cloning, chimera construction and site-directed mutagenesis

All primers used in the study are listed in Table S1. IMPDH genes were amplified from *C. neoformans* and *C. gattii* strains, cloned into pCR2.1-TOPO (Life Technologies) and sequenced. Wild-type strains utilized are listed in Table S2. *IMD1* from *C. neoformans* strain H99 and from *C. gattii* strain were subcloned into pBluescript SK- (Agilent, Santa Clara CA), then pPZP-NEO (containing a G418 resistance cassette) and pPZP-NAT (containing a nourseothricin resistance cassette) to create complementation constructs [81]. Complementation constructs for *C. neoformans* bearing the *E. coli* xanthine-guanine phosphoribosyltransferase-encoding *gpt* and *E. coli* IMP dehydrogenase-encoding *guaB* genes were generated *via* overlap PCR using the *C. neoformans ACT1* promoter and *TRP1* terminator, combined with the *E. coli* strain K12 *gpt* or *guaB* ORF, cloned into pCR2.1-TOPO and sequenced.

Site-directed mutagenesis was used to introduce new residues into wild-type *CnIMD1*. All six variants were subcloned into pBluescript SK- and further subcloned into pPZP-NEO or directly into pPZP-NAT. Double mutants and the sextuple mutant were created using successive rounds of mutagenesis. All mutagenesis products were sequenced to confirm identity and fidelity. IMPDH gene chimeras were created by dividing the gene into thirds using two common restriction sites in *CnIMD1* and *CgIMD1* (XhoI and NcoI) and reconstructing all six possible combinations. The first fragment comprises 207 residues and contains the first two unique residues V/M55 and A/T153, the second fragment comprises 225 residues and contains only the third unique residue R/K336, and the final third comprises 111 residues and contains the final three unique residues R/E446, G/A450 and A/V500. Subsequently, all chimeras were subcloned into pLITMUS28i (New England Biolabs, Ipswich MA) before further subcloning into both pPZP-NEO and pPZP-NAT. All strains created are listed in Table S3.

### Expression, purification and crystallization of *Cryptococcus* IMPDH

In-frame cloning of the cDNA coding for CnImd1 and CgImd1 into expression vectors, heterologous expression in *E. coli*, purification of protein and crystallization parameters have been described previously [82]. For this study, purified protein in crystallization buffer was mixed with a 10-fold molar excess of the substrate IMP plus 100 µM of the inhibitor MPA dissolved in dimethylsulfoxide prior to crystallization. Large crystals were obtained in wells containing 1.9 M lithium sulfate and 0.09 M imidazole/HCl pH 6.5 with either no additive or 3.0–12.0% pentaerythritol ethoxylate (15/04 EO/OH) for CnImd1.

### Steady-state and inhibitor kinetics

All enzymatic assays contained standard IMPDH assay buffer (50 mM Tris-HCl (pH 8.0), 100 mM KCl, 3 mM EDTA, 1 mM DTT, 500 µM IMP and 250 µM NAD^+^) and were performed as described [21]. Enzymatic assays were performed at 25°C in triplicate by monitoring the production of NADH at 340 nm (ε = 6.22 mM^−1^ cm^−1^) using a Varian Cary 50 spectrophotometer (Agilent Technologies, Santa Clara CA). For *K_m_* determination, IMP and NAD^+^ concentrations were varied over a range from 62.5 µM to 5,000 µM, with fixed second substrate concentrations of 250 µM NAD^+^ and 500 µM IMP, respectively. Nonlinear fitting of the data to the Michaelis-Menton equation (Equation 1) and the uncompetitive substrate inhibition equation (Equation 2) were performed using SigmaPlot 12.0 (SysStat Software, Chicago IL). IC_50_ values for both proteins were determined using fixed concentrations of IMP (850 µM or 1,800 µM) and NAD^+^ (1,000 µM) while varying the concentration of MPA (0 nM–400 nM). The *K_m_* and *k*_cat_ values were then determined in the presence of concentrations of MPA that produced 0%, 25%, 50% and 75% inhibition. Velocity data were fitted to the uncompetitive tight-binding inhibition equation (Equation 3) and the noncompetitive/mixed tight-binding inhibition equation (Equation 4) in SigmaPlot,
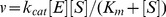
(1)

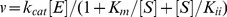
(2)

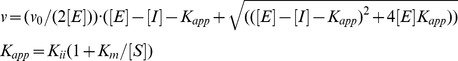
(3)

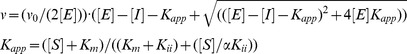
(4)where *v* is the initial velocity; *K_m_* is the Michaelis constant; *k*_cat_ is the turnover number; [*E*] is enzyme concentration; [*S*] is substrate concentration; [*I*] is inhibitor concentration; *K_ii_* and *K_is_* are the intercept and slope inhibition constants.

### Structure determination

Data were collected on the MX2 beamline of the Australian Synchrotron, Melbourne, Australia, using the *Blu-Ice* software [83]. Diffraction data were processed and reduced using *XDS* and *SCALA* [84], [85]. Initial crude molecular replacement phases were obtained using a search model based on the coordinates of the human type I IMPDH (PDB ID 1jcn) using the fast Fourier transform molecular replacement method as implemented in *Phaser* [86] as implemented in the *PHENIX* suite [87], [88]. After extensive manual re-building in *Coot* [89], combined with maximum-likelihood-based restrained refinement in *BUSTER-TNT* [90], the final model had R_work_/R_free_ values of 0.17/0.20 (Table S4). The stereochemistry of the structure was assessed and validated with *MolProbity* [91]. Data collection and refinement statistics are listed in Table S4. Figures were generated with *PyMOL* (http://www.pymol.org). Atomic coordinates and structure factors have been deposited in the Protein Data Bank, www.pdb.org (PDB ID: 4af0).

## Supporting Information

Figure S1 **Specificity of** ***Cryptococcus*** **Hpt1.** (A) The nucleobase xanthine and the nucleosides guanosine and xanthosine rescued the *imd1*Δ mutant, though with less robust growth than guanine. Neither XMP nor GMP were able to complement the auxotrophy of the *imd1*Δ strain. Hypoxanthine also did not rescue the mutant, suggesting that the *Cryptococcus* α-ketoglutarate-dependent dioxygenase is unable to perform the conversion of hypoxanthine to xanthine found in the *Schizosaccharomyces pombe* homolog. (B) All xanthylic and guanylic nucleotides rescue the MPA phenotype in the wild-type and complemented strains. Only nucleoside monophosphates after the IMPDH blockage by MPA rescue the *hpt1*Δ phenotype. (C) The *ade2*Δ mutant, defective in *de novo* purine metabolism before the pathway branchpoint, can utilize either adenine or hypoxanthine for purine nucleotide biosynthesis. Only adenine can rescue the *ade2*Δ *hpt1*Δ phenotype on minimal media. As hypoxanthine successfully supplements the purine auxotrophy of the *ade2Δ* mutant, Hpt1 can salvage hypoxanthine.
(TIF)

Figure S2 **Quantification of melanin production.** Melanin production was measured in liquid l-DOPA medium from culture supernatant at OD_475_. (A) *imd1*Δ at 30°C. (B) *imd1*Δ at 37°C. (C) *hpt1*Δ at 30°C. (D) *hpt1*Δ at 37°C. Bars represent mean OD_475_ from three replicates with standard error shown. No significant differences were found between strains.
(TIF)

Figure S3 **Effect of 20 µg/mL MPA on nematode survival.** N2 Bristol young adult nematodes were cultivated for eight days on OP50 or heat-killed OP50 on standard NGM supplemented with 6 µM 5-fluoro-2′-deoxyuridine to prevent egg laying, plus or minus 20 µg/mL MPA, the highest concentration of MPA used in the *Cryptococcus*/MPA nematode virulence assays. No significant differences were found between treatments.
(TIF)

Figure S4 **Quantitative reverse transcription PCR analysis of** ***IMD1*** **expression.** (A) Strain H99 and strain MMRL2651 were grown in YNB media at 30°C overnight and treated with 1 mM guanine before harvesting at three timepoints. (B) Strains grown overnight and treated with 5 µg/mL MPA before harvesting at three timepoints. Transcript levels of *CnIMD1* and *CgIMD1* are expressed as a fold change relative to expression of the housekeeping gene β-tubulin. Standard error bars are shown. *p*<0.05 *; *p*<0.01 **.
(TIF)

Figure S5 **Alignment of IMPDH from all eight molecular types of** ***Cryptococcus*** **.** IMPDH was amplified and sequenced from the four molecular types for which there was no existing sequence data (VNII, VNB, VGIII & VGIV) and aligned with existing sequence data for VNI, VNIV, VGI and VGII. MPA-resistant *C. gattii* VGIV IMPDH has 22 substitutions compared to MPA-sensitive *C. neoformans* VNI IMPDH, although only six of these residues are not shared with another molecular type. Residues unique to VGIV are highlighted in red. The IMPDH accessory domain is highlighted in green, the active site loop in blue and the mobile flap in orange.
(TIF)

Figure S6 **Growth of all IMPDH mutants on MPA.** Serial dilution spotting assays of all IMPDH mutants on YNB plus 5 µg/mL MPA. The juxtaposed images depict which portions of the IMPDH allele are present, with yellow representing *CnIMD1* and blue representing *CgIMD1*, split into three thirds with the vertical bars depicting the six unique residues. Two proteins are depicted for the H99 background where the wild-type allele is also present. (A) Transformation of IMPDH variants (point mutants, double mutants, sextuple mutants, chimeras plus the two wild-type alleles) into the *imd1*Δ deletion background. (B) Transformation of IMPDH variants (point mutants, double mutants, sextuple mutants, chimeras plus the two wild-type alleles) into the wild-type H99 background, which possesses one copy of the MPA-sensitive allele.
(TIF)

Figure S7 **Steady-state kinetics of** ***Cryptococcus*** **IMPDH.** Plots of velocity *versus* IMP and velocity *versus* NAD^+^ concentration were generated by fixing one substrate (250 µM IMP and 500 µM NAD^+^) and varying the other. For both enzymes, velocity *versus* IMP plots were best described by the Michaelis-Menton equation, while velocity *versus* NAD^+^ plots were best fit by the uncompetitive substrate inhibition equation. (A) CnImd1. (B) CgImd1.
(TIF)

Figure S8 **Inhibitor kinetics of** ***Cryptococcus*** **IMPDH.** Inhibition by MPA was investigated at fixed concentration of IMP (850 µM for CnImd1 and 1,800 µM for CgImd1) and varying NAD^+^ (62.5 µM–2,000 µM) and indicated MPA concentrations. Initial velocity data was best described by the uncompetitive tight-binding inhibition equation *versus* NAD^+^, while initial velocity data of MPA *versus* IMP were best fit by a noncompetitive/mixed tight-binding model. (A) CnImd1. (B) CgImd1.
(TIF)

Table S1 Primers used in this study.
(DOC)

Table S2 Wild-type strains used in this study.
(DOC)

Table S3 Strains created for this study.
(DOC)

Table S4 Crystallographic data collection and refinement statistics.
(DOC)
