# Supplementary figures and images for: De novo GTP Biosynthesis Is Critical for Virulence of the Fungal Pathogen Cryptococcus neoformans
Source: PLoS Pathog. 2012 Oct 11;8(10):e1002957. doi: 10.1371/journal.ppat.1002957 (PMC3469657; doi:10.1371/journal.ppat.1002957)

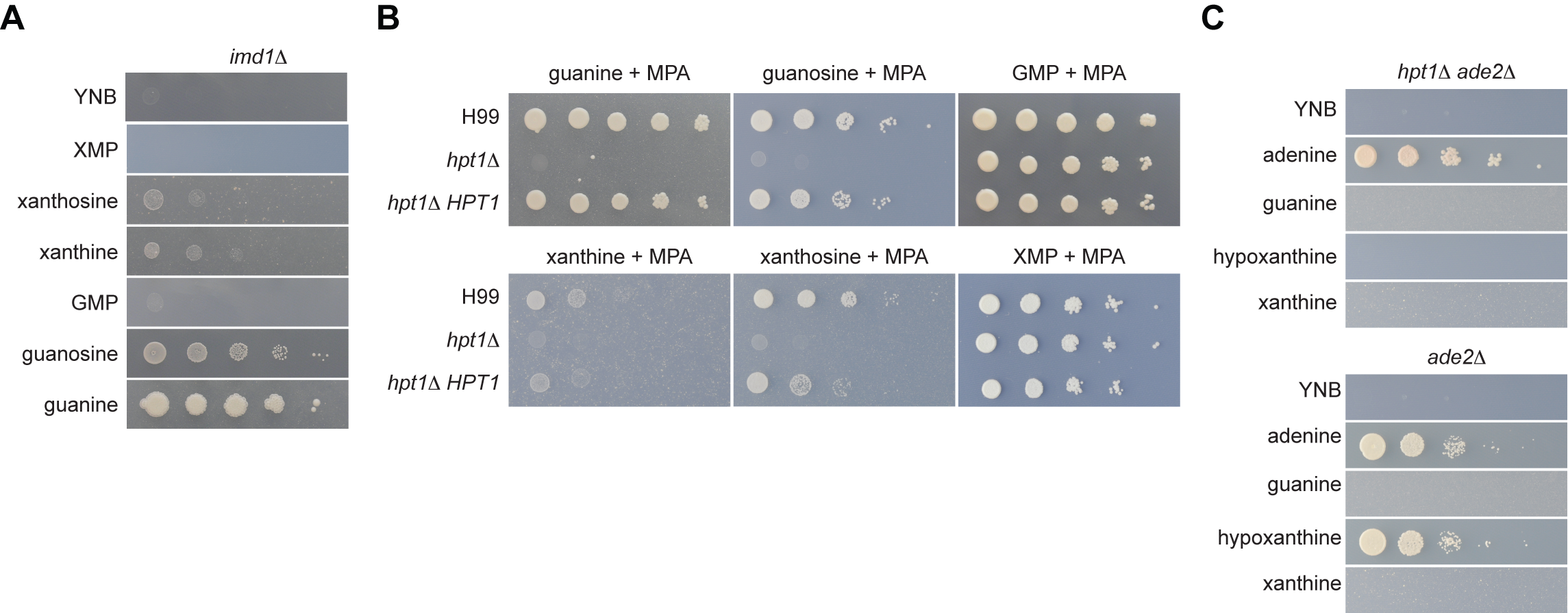

Supplement: Figure S1 — Specificity of Cryptococcus Hpt1. (A) The nucleobase xanthine and the nucleosides guanosine and xanthosine rescued the imd1Δ mutant, though with less robust growth than guanine. Neither XMP nor GMP were able to complement the auxotrophy of the imd1Δ strain. Hypoxanthine also did not rescue the mutant, suggesting that the Cryptococcus α-ketoglutarate-dependent dioxygenase is unable to perform the conversion of hypoxanthine to xanthine found in the Schizosaccharomyces pombe homolog. (B) All xanthylic and guanylic nucleotides rescue the MPA phenotype in the wild-type and complemented strains. Only nucleoside monophosphates after the IMPDH blockage by MPA rescue the hpt1Δ phenotype. (C) The ade2Δ mutant, defective in de novo purine metabolism before the pathway branchpoint, can utilize either adenine or hypoxanthine for purine nucleotide biosynthesis. Only adenine can rescue the ade2Δ hpt1Δ phenotype on minimal media. As hypoxanthine successfully supplements the purine auxotrophy of the ade2Δ mutant, Hpt1 can salvage hypoxanthine. (TIF) [file ppat.1002957.s001.tif]

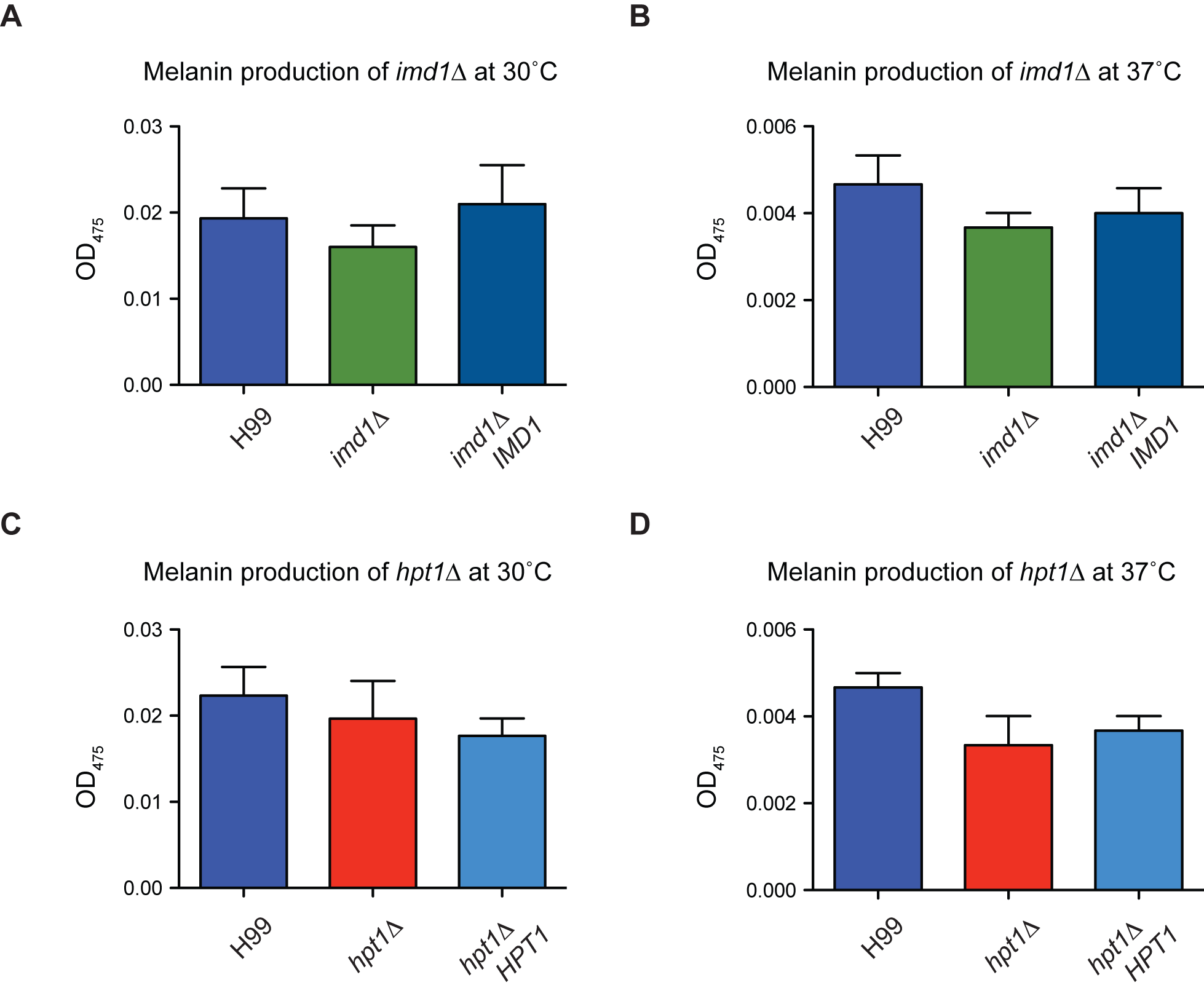

Supplement: Figure S2 — Quantification of melanin production. Melanin production was measured in liquid l-DOPA medium from culture supernatant at OD475. (A) imd1Δ at 30°C. (B) imd1Δ at 37°C. (C) hpt1Δ at 30°C. (D) hpt1Δ at 37°C. Bars represent mean OD475 from three replicates with standard error shown. No significant differences were found between strains. (TIF) [file ppat.1002957.s002.tif]

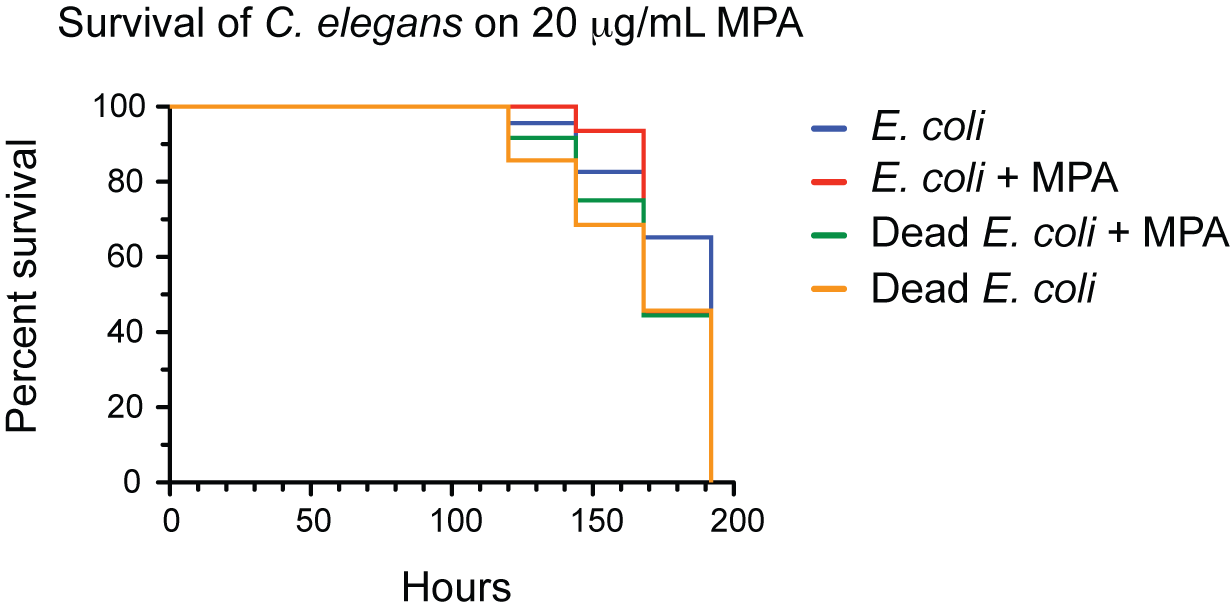

Supplement: Figure S3 — Effect of 20 µg/mL MPA on nematode survival. N2 Bristol young adult nematodes were cultivated for eight days on OP50 or heat-killed OP50 on standard NGM supplemented with 6 µM 5-fluoro-2′-deoxyuridine to prevent egg laying, plus or minus 20 µg/mL MPA, the highest concentration of MPA used in the Cryptococcus/MPA nematode virulence assays. No significant differences were found between treatments. (TIF) [file ppat.1002957.s003.tif]

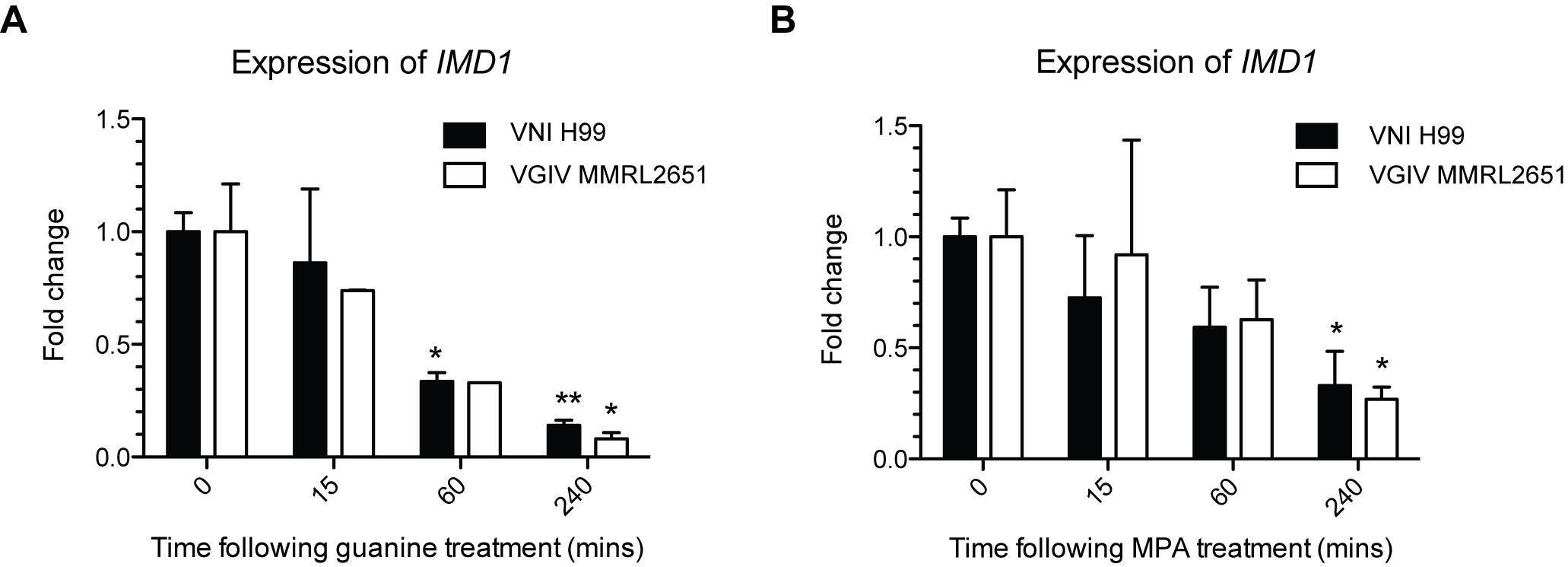

Supplement: Figure S4 — Quantitative reverse transcription PCR analysis of IMD1 expression. (A) Strain H99 and strain MMRL2651 were grown in YNB media at 30°C overnight and treated with 1 mM guanine before harvesting at three timepoints. (B) Strains grown overnight and treated with 5 µg/mL MPA before harvesting at three timepoints. Transcript levels of CnIMD1 and CgIMD1 are expressed as a fold change relative to expression of the housekeeping gene β-tubulin. Standard error bars are shown. p<0.05 *; p<0.01 **. (TIF) [file ppat.1002957.s004.tif]

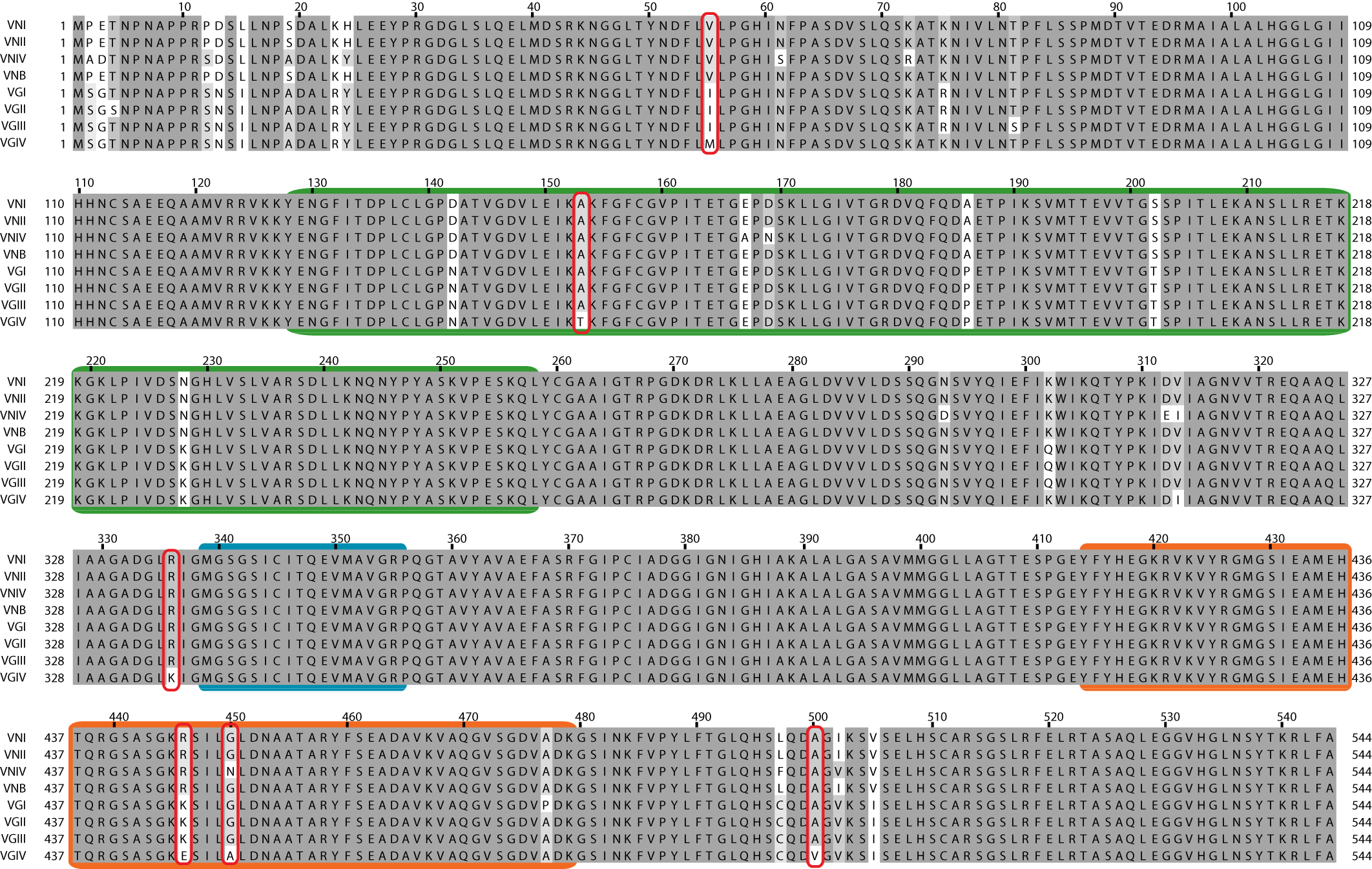

Supplement: Figure S5 — Alignment of IMPDH from all eight molecular types of Cryptococcus . IMPDH was amplified and sequenced from the four molecular types for which there was no existing sequence data (VNII, VNB, VGIII & VGIV) and aligned with existing sequence data for VNI, VNIV, VGI and VGII. MPA-resistant C. gattii VGIV IMPDH has 22 substitutions compared to MPA-sensitive C. neoformans VNI IMPDH, although only six of these residues are not shared with another molecular type. Residues unique to VGIV are highlighted in red. The IMPDH accessory domain is highlighted in green, the active site loop in blue and the mobile flap in orange. (TIF) [file ppat.1002957.s005.tif]

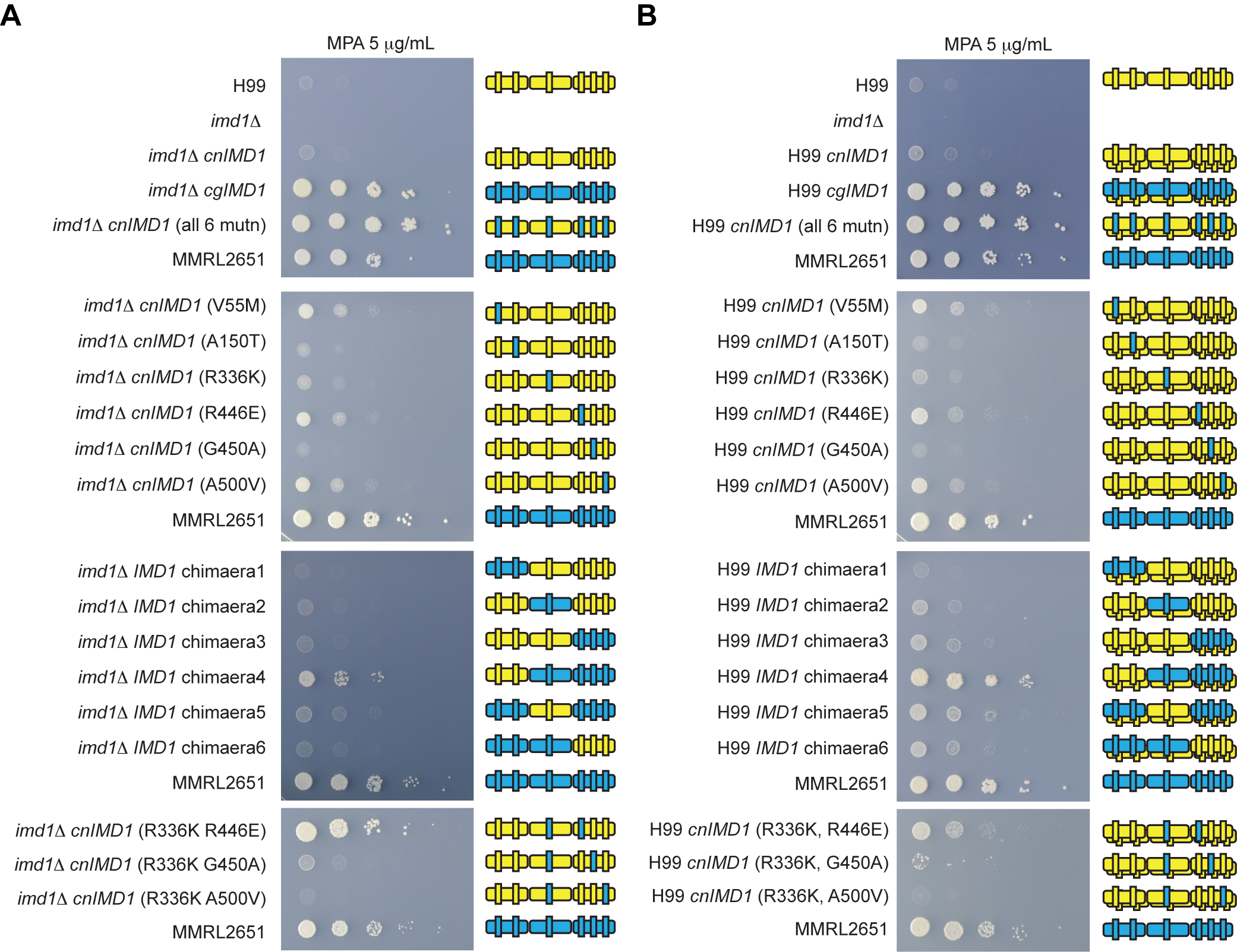

Supplement: Figure S6 — Growth of all IMPDH mutants on MPA. Serial dilution spotting assays of all IMPDH mutants on YNB plus 5 µg/mL MPA. The juxtaposed images depict which portions of the IMPDH allele are present, with yellow representing CnIMD1 and blue representing CgIMD1, split into three thirds with the vertical bars depicting the six unique residues. Two proteins are depicted for the H99 background where the wild-type allele is also present. (A) Transformation of IMPDH variants (point mutants, double mutants, sextuple mutants, chimeras plus the two wild-type alleles) into the imd1Δ deletion background. (B) Transformation of IMPDH variants (point mutants, double mutants, sextuple mutants, chimeras plus the two wild-type alleles) into the wild-type H99 background, which possesses one copy of the MPA-sensitive allele. (TIF) [file ppat.1002957.s006.tif]

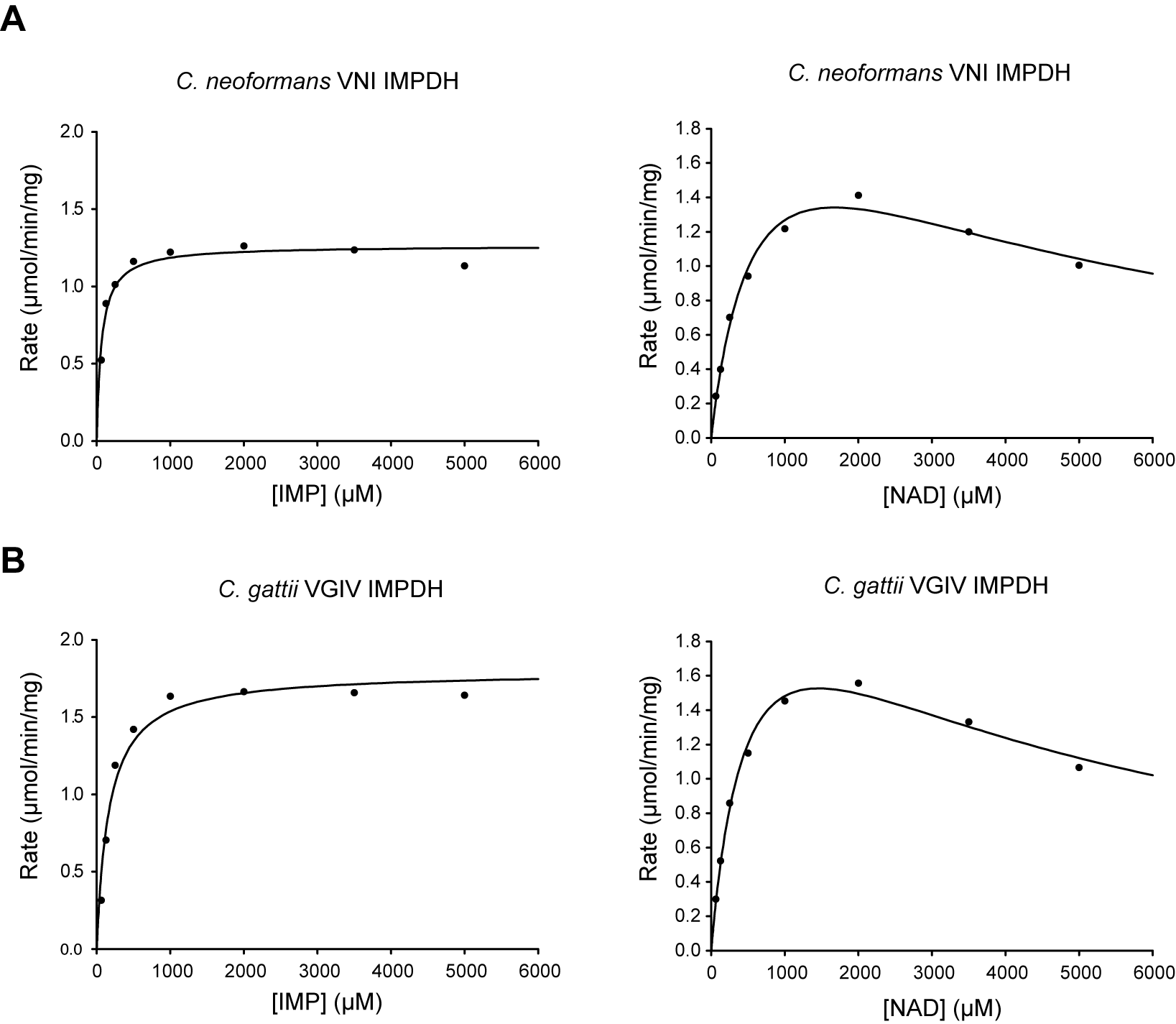

Supplement: Figure S7 — Steady-state kinetics of Cryptococcus IMPDH. Plots of velocity versus IMP and velocity versus NAD+ concentration were generated by fixing one substrate (250 µM IMP and 500 µM NAD+) and varying the other. For both enzymes, velocity versus IMP plots were best described by the Michaelis-Menton equation, while velocity versus NAD+ plots were best fit by the uncompetitive substrate inhibition equation. (A) CnImd1. (B) CgImd1. (TIF) [file ppat.1002957.s007.tif]

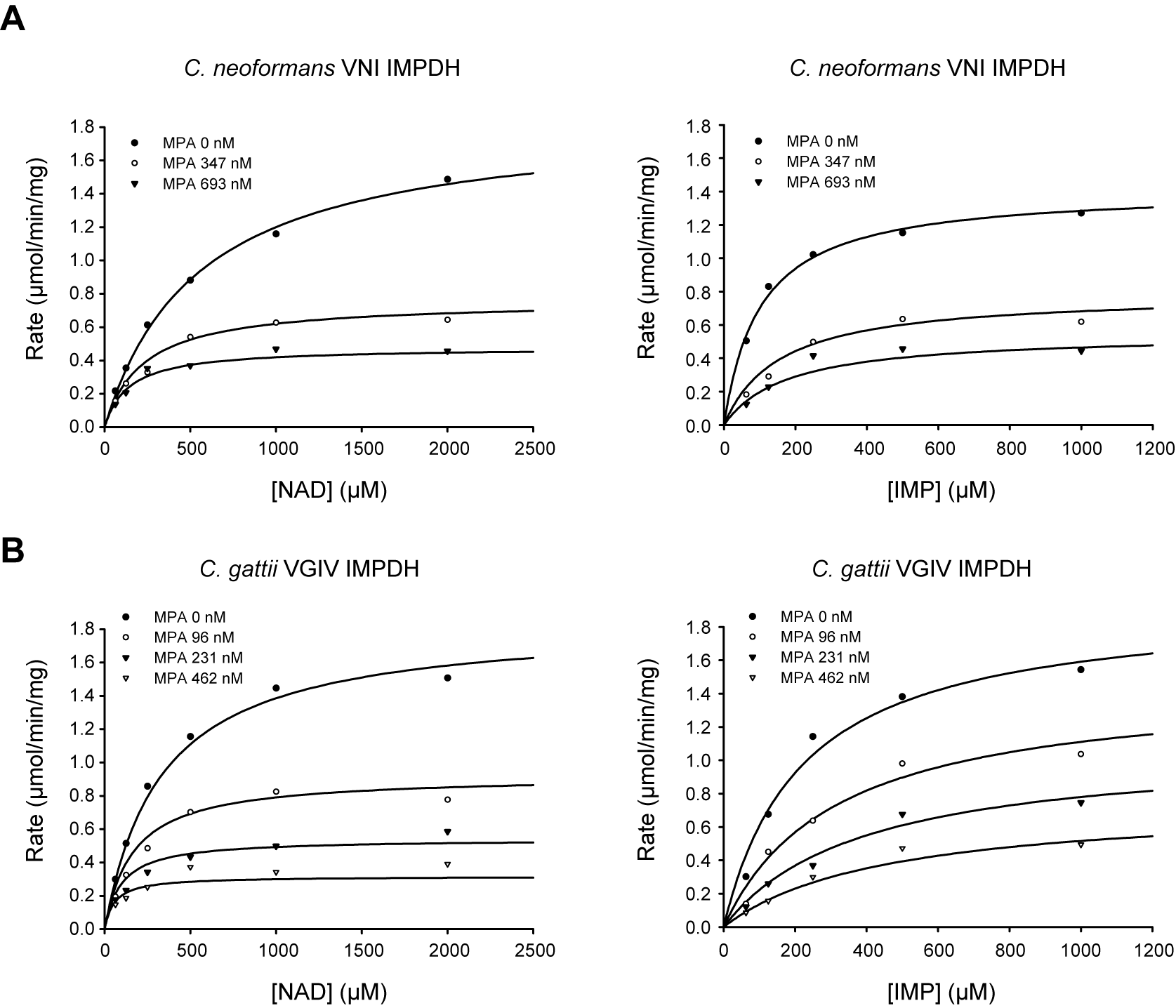

Supplement: Figure S8 — Inhibitor kinetics of Cryptococcus IMPDH. Inhibition by MPA was investigated at fixed concentration of IMP (850 µM for CnImd1 and 1,800 µM for CgImd1) and varying NAD+ (62.5 µM–2,000 µM) and indicated MPA concentrations. Initial velocity data was best described by the uncompetitive tight-binding inhibition equation versus NAD+, while initial velocity data of MPA versus IMP were best fit by a noncompetitive/mixed tight-binding model. (A) CnImd1. (B) CgImd1. (TIF) [file ppat.1002957.s008.tif]
