# Supplementary material for: De novo GTP Biosynthesis Is Critical for Virulence of the Fungal Pathogen Cryptococcus neoformans
Source: PLoS Pathog. 2012 Oct 11;8(10):e1002957. doi: 10.1371/journal.ppat.1002957 (PMC3469657; doi:10.1371/journal.ppat.1002957)
Supplement: Table S1 — Primers used in this study. (DOC) [file ppat.1002957.s009.doc]

**Table S1: Primers used in this study**

| **Primer name** | **Purpose** | **Sequence** |
| --- | --- | --- |
|  |  |  |
| UQ399 | *IMD1* upper | CCATGTCATGCTGCTTGCTTG |
| UQ261 | *IMD1* lower | GTACGGAAAATGATCGAGGAA |
| UQ299 | *IMD1* sequencing 1 | GGCTTCTGCGGTGTTCCTATC |
| UQ300 | *IMD1* sequencing 2 | TACCGCTGTGTACGCCGTTGC |
| UQ301 | *IMD1* sequencing 3 | AAGAAGACCGCCCATCATAAC |
| UQ302 | *IMD1* sequencing 4 | ACGAGCCGGTGACAACTTCAG |
| UQ262 | *ACT1* promoter upper | GCTGCGAGGATGTGAGCTGGA |
| UQ263 | *gpt*/*ACT1* promoter lower | GATGTATTTTTCGCTCATAGACATGTTGGGCGAGTT |
| UQ264 | *gpt* upper | AACTCGCCCAACATGTCTATGAGCGAAAAATACATC |
| UQ265 | *gpt* lower | ACCCCTTACCGCCTTCACTTAGCGACCGGAGATTGG |
| UQ266 | *gpt*/*TRP1* terminator upper | CCAATCTCCGGTCGCTAAGTGAAGGCGGTAAGGGGT |
| UQ267 | *TRP1* terminator lower | GGTTTATCTGTATTAACACGG |
| UQ375 | *guaB*/*ACT1* promoter lower | TTTAGCGATACGTAGCATAGACATGTTGGGCGAGTT |
| UQ376 | *guaB* upper | AACTCGCCCAACATGTCTATGCTACGTATCGCTAAA |
| UQ377 | *guaB* lower | ACCCCTTACCGCCTTCACTCAGGAGCCCAGACGGTA |
| UQ378 | *guaB*/*TRP1* terminator upper | TACCGTCTGGGCTCCTGAGTGAAGGCGGTAAGGGGT |
| JOHE9392 | *NEO*/*NAT* marker upper | GCTGCGAGGATGTGAGCTGGAGAGCG |
| JOHE9393 | *NEO*/*NAT* marker lower | GGTTTATCTGTATTAACACGGAAGAGATGTAG |
| JOHE8994 | KO tester 5' | TGTGGATGCTGGCGGAGGATA |
| UQ1269 | KO tester 3' | GAGACAGACATCGTGTCAATC |
| UQ580 | *IMD1* KO 5' upper | GTTTCGGGTGGAGTGAGGTCT |
| UQ581 | *IMD1* KO 5' lower | TCCAGCTCACATCCTCGCAGCTATTACAAAGTCGTAGGGGGG |
| UQ582 | *IMD1* KO 3' upper | CCGTGTTAATACAGATAAACCACATAGAGAGATGTGGGAGTA |
| UQ583 | *IMD1* KO 3' lower | GACAGGCGCTTGAGAAAGAAG |
| UQ1059 | *IMD1* KO tester 5' | GAACCTCTTTGCTTGCCGTTT |
| UQ1272 | *IMD1* KO tester 3' | ATCGGGCGTTACTTCATCTCA |
| UQ584 | *HPT1* KO 5' upper | CCGGAATTGTTGAAGTTCGTA |
| UQ585 | *HPT1* KO 5' lower | TCCAGCTCACATCCTCGCAGCGGTGTATAGTTTTAAACAGAT |
| UQ586 | *HPT1* KO 3' upper | CCGTGTTAATACAGATAAACCGCCGTTTCCCTGCTCGTTCTT |
| UQ587 | *HPT1* KO 3' lower | CGTTCAAACGTCAGCTCTGTC |
| UQ710 | *HPT1* seq primer upper | ATTACTTCCCGGCAAAGCAGT |
| UQ711 | *HPT1* seq primer lower | GGGTGAGGGGAGTTTGTGTGG |
| UQ1439 | *ADE2* KO 5' upper | GAGTTAAAGTGTCGATGGCAG |
| UQ1440 | *ADE2* KO 5' lower | CCAGCTCACATCCTCGCAGCTTTGCTACAAGGGGTGCGGATG |
| UQ1441 | *ADE2* KO 3' upper | CCGTGTTAATACAGATAAACCATTGGTGCGATATCTGTAACT |
| UQ1442 | *ADE2* KO 3' lower | CGCTTAGGACAAGAGAGGCTA |
| UQ484 | *TUB2* qRT-PCR upper | AGTCGCTTTTCAAGCGTATCG |
| UQ729 | *TUB2* qRT-PCR lower | GGATTCGGCTTCAGAGAATTCA |
| UQ1513 | *CnIMD1* qRT-PCR upper | GCCAGCCGATTTGGGATT |
| UQ1514 | *CnIMD1* qRT-PCR lower | AACGGCAGAAGCACCAAGAG |
| UQ1507 | *CgIMD1* qRT-PCR upper | GCCAGCCGATTTGGGATT |
| UQ1508 | *CgIMD1* qRT-PCR lower | ACCCATCATGACGGCAGAAG |
| UQ547 | H99 *IMD1* R446E mutagenesis lower 5' | CCGAGGATAGACTCCTTGCCGGAAGC |
| UQ548 | H99 *IMD1* R446E mutagenesis upper 3' | GCTTCCGGCAAGGAGTCTATCCTCGG |
| UQ576 | H99 *IMD1* V55M mutagenesis upper | AATGACTTCTTGATGCTTCCTGGACAC |
| UQ577 | H99 *IMD1* V55M mutagenesis lower | GTGTCCAGGAAGCATCAAGAAGTCATT |
| UQ578 | H99 *IMD1* A153T mutagenesis upper | CTTGAGATCAAGACTAAGTTCGGCT |
| UQ579 | H99 *IMD1* A153T mutagenesis lower | AGCCGAACTTAGTCTTGATCTCAAG |
| UQ813 | H99 *IMD1* R336K mutagenesis upper | CCGATGGCTTGAAGATTGGTATGGG |
| UQ814 | H99 *IMD1* R336K mutagenesis lower | CCCATACCAATCTTCAAGCCATCGG |
| UQ815 | H99 *IMD1* G450A mutagenesis upper | GGTCTATCCTCGCTTTGGACAACGC |
| UQ816 | H99 *IMD1* G450A mutagenesis lower | GCGTTGTCCAAAGCGAGGATAGACC |
| UQ817 | H99 *IMD1* A500V mutagenesis upper | CTCTCCAGGATGTTGGTATCAAGAGG |
| UQ818 | H99 *IMD1* A500V mutagenesis lower | CCTCTTGATACCAACATCCTGGAGAG |
| UQ1066 | H99 *IMD1* R446E G450A mutagenesis upper | GGCAAGGAGTCTATCCTCGCTTTGGA |
| UQ1067 | H99 *IMD1* R446E G450A mutagenesis lower | TCCAAAGCGAGGATAGACTCCTTGCC |
| UQ615 | H99 *IMD1* cDNA upper | TAATAATGCCTGAAACCAACC |
| UQ616 | H99 *IMD1* cDNA lower | TCTCTCTATGTCTAGGCGAAC |
| UQ648 | MMRL2651 *IMD1* cDNA upper | TAATAATGTCTGGCACCAACC |
| UQ649 | MMRL2651 *IMD1* cDNA lower | CTTGTTTAGGCGAACAGTCTC |
| UQ928 | pQE-30 F sequencing primer | CGGATAACAATTTCACACAG |
| UQ929 | pQE-30 R sequencing primer | GTTCTGAGGTCATTACTGG |
|  |  |  |
