# Supplementary material for: De novo GTP Biosynthesis Is Critical for Virulence of the Fungal Pathogen Cryptococcus neoformans
Source: PLoS Pathog. 2012 Oct 11;8(10):e1002957. doi: 10.1371/journal.ppat.1002957 (PMC3469657; doi:10.1371/journal.ppat.1002957)
Supplement: Table S2 — Wild-type strains used in this study. (DOC) [file ppat.1002957.s010.doc]

**Table S2: Wild-type strains used in this study**

| **Strain** | **Species** | **Molecular Type** | **Mating Type** |
| --- | --- | --- | --- |
|  |  |  |  |
| H99 | *Cryptococcus neoformans* var. *grubii* | VNI | *MAT*α |
| 125.91 | *Cryptococcus neoformans* var. *grubii* | VNI | *MAT***a** |
| KN99a | *Cryptococcus neoformans* var. *grubii* | VNI | *MAT***a** |
| 8.1 | *Cryptococcus neoformans* var. *grubii* | VNII | *MAT*α |
| I57 | *Cryptococcus neoformans* var. *grubii* | VNII | *MAT*α |
| JEC21 | *Cryptococcus neoformans* var. *neoformans* | VNIV | *MAT*α |
| NIH430 | *Cryptococcus neoformans* var. *neoformans* | VNIV | *MAT***a** |
| Bt33 | *Cryptococcus neoformans* var. *grubii* | VNB | *MAT*α |
| Bt63 | *Cryptococcus neoformans* var. *grubii* | VNB | *MAT***a** |
| WM276 | *Cryptococcus gattii* | VGI | *MAT*α |
| E566 | *Cryptococcus gattii* | VGI | *MAT***a** |
| R265 | *Cryptococcus gattii* | VGII | *MAT*α |
| CBS1930 | *Cryptococcus gattii* | VGII | *MAT***a** |
| NIH312 | *Cryptococcus gattii* | VGIII | *MAT*α |
| B4546 | *Cryptococcus gattii* | VGIII | *MAT***a** |
| MMRL2651 | *Cryptococcus gattii* | VGIV | *MAT*α |
| Bt201 | *Cryptococcus gattii* | VGIV | *MAT*α |
| ATCC 90113 | *Cryptococcus neoformans* var. *grubii* | VNI | *MAT*α |
| ATCC 90028 | *Candida albicans* | NA | NA |
| ATCC 6258 | *Candida krusei* | NA | NA |
| ATCC 22019 | *Candida parapsilosis* | NA | NA |
|  |  |  |  |
