# Supplementary material for: De novo GTP Biosynthesis Is Critical for Virulence of the Fungal Pathogen Cryptococcus neoformans
Source: PLoS Pathog. 2012 Oct 11;8(10):e1002957. doi: 10.1371/journal.ppat.1002957 (PMC3469657; doi:10.1371/journal.ppat.1002957)
Supplement: Table S3 — Strains created for this study. (DOC) [file ppat.1002957.s011.doc]

**Table S3: Strains created for this study**

| **Strain** | **Genotype** | **Molecular Type** | **Mating Type** |
| --- | --- | --- | --- |
|  |  |  |  |
| CM6 | *imd1∆ NEO* | VNI | *MAT*α |
| CM129 | *imd1∆ NEO CnIMD1 NAT* | VNI | *MAT*α |
| CM25 | *imd1∆ NEO guaB NAT* | VNI | *MAT*α |
| CM298 | *imd1∆ NAT* | VNI | *MAT***a** |
| CM1 | *hpt1∆ NEO* | VNI | *MAT*α |
| CM131 | *hpt1∆ NEO HPT1 NAT* | VNI | *MAT*α |
| CM23 | *hpt1∆ NEO gpt NAT* | VNI | *MAT*α |
| M049 | *ade2∆* | VNI | *MAT*α |
| CM285 | *hpt1∆ NEO ade2∆ NAT* | VNI | *MAT*α |
| CM56 | *CnIMD1 NAT* | VNI | *MAT*α |
| CM88 | *CgIMD1 NAT* | VNI | *MAT*α |
| CM280 | *imd1∆ NEO CgIMD1 NAT* | VNI | *MAT*α |
| CM97 | *imd1∆* *NEO CnIMD1* V55M *NAT* | VNI | *MAT*α |
| CM99 | *imd1∆* *NEO CnIMD1* A153T *NAT* | VNI | *MAT*α |
| CM283 | *imd1∆* *NEO CnIMD1* R336K *NAT* | VNI | *MAT*α |
| CM282 | *imd1∆* *NEO CnIMD1* R446E *NAT* | VNI | *MAT*α |
| CM244 | *imd1∆* *NEO CnIMD1* G450A *NAT* | VNI | *MAT*α |
| CM246 | *imd1∆* *NEO CnIMD1* A500V *NAT* | VNI | *MAT*α |
| CM59 | *CnIMD1* V55M *NAT* | VNI | *MAT*α |
| CM61 | *CnIMD1* A153T *NAT* | VNI | *MAT*α |
| CM63 | *CnIMD1* R336K *NAT* | VNI | *MAT*α |
| CM58 | *CnIMD1* R446E *NAT* | VNI | *MAT*α |
| CM65 | *CnIMD1* G450A *NAT* | VNI | *MAT*α |
| CM67 | *CnIMD1* A500V *NAT* | VNI | *MAT*α |
| CM200 | *imd1∆* *NEO CgIMD1*/*CnIMD1*/*CnIMD1* chimaera *NAT* | VNI | *MAT*α |
| CM248 | *imd1∆* *NEO CnIMD1*/*CgIMD1*/*CnIMD1* chimaera *NAT* | VNI | *MAT*α |
| CM250 | *imd1∆* *NEO CnIMD1*/*CnIMD1*/*CgIMD1* chimaera *NAT* | VNI | *MAT*α |
| CM188 | *imd1∆* *NEO CgIMD1*/*CgIMD1*/*CnIMD1* chimaera *NAT* | VNI | *MAT*α |
| CM254 | *imd1∆* *NEO CgIMD1*/*CnIMD1*/*CgIMD1* chimaera *NAT* | VNI | *MAT*α |
| CM252 | *imd1∆* *NEO CnIMD1*/*CgIMD1*/*CgIMD1* chimaera *NAT* | VNI | *MAT*α |
| CM35 | *CnIMD1*/*CnIMD1*/*CgIMD1* chimaera *NAT* | VNI | *MAT*α |
| CM41 | *CnIMD1*/*CgIMD1*/*CnIMD1* chimaera *NAT* | VNI | *MAT*α |
| CM78 | *CgIMD1*/*CnIMD1*/*CnIMD1* chimaera *NAT* | VNI | *MAT*α |
| CM42 | *CgIMD1*/*CgIMD1*/*CnIMD1* chimaera *NAT* | VNI | *MAT*α |
| CM43 | *CgIMD1*/*CnIMD1*/*CgIMD1* chimaera *NAT* | VNI | *MAT*α |
| CM39 | CnIMD1/*CgIMD1*/*CgIMD1* chimaera *NAT* | VNI | *MAT*α |
| CM209 | *imd1∆ NEO CnIMD1* V55M A153T R336K R446E G450A A500V *NAT* | VNI | *MAT*α |
| CM82 | *CnIMD1* V55M A153T R336K R446E G450A A500V *NAT* | VNI | *MAT*α |
| CM302 | *imd1∆ NEO CnIMD1* R336K R446E *NAT* | VNI | *MAT*α |
| CM303 | *imd1∆ NEO CnIMD1* R336K G450A *NAT* | VNI | *MAT*α |
| CM304 | *imd1∆ NEO CnIMD1* R336K A500V *NAT* | VNI | *MAT*α |
| CM299 | *CnIMD1* R336K R446E *NAT* | VNI | *MAT*α |
| CM300 | *CnIMD1* R336K G450A *NAT* | VNI | *MAT*α |
| CM301 | *CnIMD1* R336K A500V *NAT* | VNI | *MAT*α |
|  |  |  |  |
