# Supplementary material for: De novo GTP Biosynthesis Is Critical for Virulence of the Fungal Pathogen Cryptococcus neoformans
Source: PLoS Pathog. 2012 Oct 11;8(10):e1002957. doi: 10.1371/journal.ppat.1002957 (PMC3469657; doi:10.1371/journal.ppat.1002957)
Supplement: Table S4 — Crystallographic data collection and refinement statistics. (DOC) [file ppat.1002957.s012.doc]

**Table S4:** **Crystallographic** **data collection and refinement statistics**

| **Data collection statistics** |  |
| --- | --- |
| Wavelength (Å) | 0.97 |
| Data collection temperature (K) | 100 |
| Space group | *I*4 |
| Unit cell parameters: a, b, c (Å); α, β, γ (°) | 149.6, 149.6, 122.7; 90, 90, 90 |
| Resolution range (outer shell in brackets; Å) | 47.4 – 2.20 (2.26 – 2.20) |
| Unique reflections | 67,847 |
| Total observations | 351,693 |
| <I / σ(I)>: all (outer shell) | 22.1 (1.8) |
| R_merge_: all (outer shell)^*^ | 3.4 (95.6) |
| Completeness: all (outer shell) (%) | 99.1 (99.7) |
| Multiplicity | 5.9 |
| Wilson B-factor | 33.5 |
| **Refinement statistics** |  |
| Amino acid residues | 4-129, 245-437, 450-525 |
| Non-hydrogen protein atoms | 5,909 |
| Non-protein ligands | 2 × MOA, 2 × IMP |
| Solvent content (%) | 63.5 |
| Bond length deviation from ideal values (Å) | 0.01 |
| Bond angle deviation from ideal values (°) | 1.12 |
| Peptide omega torsion angles (°) | 3.02 |
| Other torsion angles (°) | 17.2 |
| Average B-factor (Å^2^) | 40.2 |
| Ramachandran favored/outliers (%) | 96.4/0.1 |
| All-atom clashscore (97^th^ percentile^*^) | 7.0 |
| Random reflections assigned for cross-validation | 3,429 |
| R_work_/R_free_ (%)^†^ | 17.3/20.8 |
| MolProbity score^‡^ (92^nd^ percentile^*^) | 1.84 |
| Luzzati plot estimate of the coordinate error (Å) | 0.24 |

^*^R_merge_ = ∑*_hkl_*(∑*_i_*(|I*_hkl,i_*-<I*_hkl_*>|))/∑*_hkl,i_* <I*_hkl_*>, where I *_hkl,i_* is the intensity of an individual measurement of the reflection with Miller indices h, k and l, and <I_hkl_> is the mean intensity of that reflection. Calculated for I > -3σ(I).

^†^R_work_ = ∑*_hkl_*(||Fobs*_hkl_*|-|Fcalc*_hkl_*||)/|Fobs*_hkl_*|, where |Fobs*_hkl_*| and |Fcalc*_hkl_*| are the observed and calculated structure factor amplitudes. R_free_ is equivalent to R_work_ but calculated with reflections (5%) omitted from the refinement process.

^‡^MolProbity score is defined as follows: 0.42574*log(1+clashscore)+0.32996*log(1+max(0,pctRotOut-1))+0.24979*log(1+max(0,100-pctRamaFavored-2))+0.5; ^*^100^th^ percentile is the best among structures of comparable resolution; 0^th^ percentile is the worst.
